# Exploring the protective effect of metformin against sarcopenia: insights from cohort studies and genetics

**DOI:** 10.1186/s12967-025-06357-x

**Published:** 2025-03-21

**Authors:** Yanyan Hu, Shan Lu, Cheng Xue, Zhaonian Hu, Yifei Wang, Wensong Zhang, Dan Wang, Jizheng Wang, Guoxian Ding, Jing Yu, Yifang Hu, Yun Liu

**Affiliations:** 1 Department of Geriatrics, The First Affiliated Hospital of Nanjing Medical University, Nanjing, Jiangsu China; 2 Women and Children Department of the First Affiliated Hospital of Nanjing Medical University, Nanjing, Jiangsu China; 3 Department of Psychology, School of Social and Behavioral Sciences, Nanjing University, Nanjing, Jiangsu China; 4 Department of the Core Facility, The First Affiliated Hospital of Nanjing Medical University, Nanjing, Jiangsu China; 5 Department of the Clinical Medical Research, The Friendship Hospital of Ili Kazakh Autonomous Prefecture, Yining, Xinjiang China

**Keywords:** Metformin, Sarcopenia, NHANES, Mendelian randomization, Molecular docking

## Abstract

**Background:**

The impact of metformin on sarcopenia remains uncertain. This study aimed to investigate whether metformin influences sarcopenia risk and evaluate the effects of potential drug targets on sarcopenia traits.

**Methods:**

We analyzed data from the National Health and Nutrition Examination Survey (NHANES) (n = 3549) to assess the association between metformin use and sarcopenia risk in elderly patients with type 2 diabetes. Mendelian randomization (MR) analysis using genome-wide association studies (GWAS) from UK Biobank (n = 1,366,167) and FinnGen (n = 218,007), with expression quantitative trait loci (eQTL) as instrumental variables, examined the causal effect of metformin-related targets on sarcopenia traits, while molecular docking explored the interaction between metformin and its drug targets.

**Results:**

Metformin use was associated with increased grip strength (OR = 2.46; 95% CI 1.49–2.38) and skeletal muscle mass (OR = 1.24; 95% CI 0.20–2.28), as well as reduced mortality (HR = 0.62; 95% CI 0.54–0.71). MR analysis suggested a possible link between GDF15 gene expression and sarcopenia traits, with no evidence of genetic confounding. Molecular docking indicated stable binding between metformin and GDF15.

**Conclusion:**

This study suggests that metformin may lower sarcopenia risk, particularly in elderly patients with type 2 diabetes, with GDF15 identified as a promising target for sarcopenia treatment.

**Supplementary Information:**

The online version contains supplementary material available at 10.1186/s12967-025-06357-x.

## Introduction

Sarcopenia is defined as a syndrome characterized by age-related loss of muscle strength, muscle mass, or physical function, which can have serious adverse consequences such as fractures and falls, dysphagia, and even death [1]. Type 2 diabetes mellitus (T2DM) is one of the most prevalent metabolic diseases and has a significant impact on mortality. Sarcopenia is 1.5 times more prevalent in older T2DM patients, with an accelerated loss of muscle strength and skeletal muscle content [2, 3]. Many studies have also shown that older people with T2DM have slower walking speed and an increased risk of poor homeostasis, which has a significant negative impact on physical function and quality of life [4]. A recently Mendelian randomization (MR) study has demonstrated a bi-directional causal relationship between sarcopenia and diabetes [5]. Morever, European working group has proposed the concept of “diabetic sarcopenia” as a novel complication of diabetes, which raises new challenges and concerns for their prevention and treatment [6, 7]. Currently, there is no specific, highly effective treatment for sarcopenia, making it even more critical to develop targeted therapeutic approaches for patients affected by diabetic sarcopenia [8, 9].

Emerging evidence suggests that insulin resistance plays a significant role in the pathogenesis of sarcopenia, and pharmacological treatments targeting glucose metabolism, such as metformin, may hold promise for mitigating sarcopenia [10]. Metformin, a well-established hypoglycemic agent, enhances insulin sensitivity and exerts antioxidant and anti-inflammatory effects, which could potentially benefit skeletal muscle mass and function [11]. Metformin has been shown to restore autophagic flux and mitochondrial function in myoblasts, which are critical for preventing age-related muscle loss [12]. Furthermore, a combination of metformin and galantamine has shown synergistic effects in treating sarcopenia, providing an additional therapeutic approach [13]. Studies also indicate that metformin could reduce frailty and improve muscle function in older adults, particularly those with hypertension and prediabetes [14–16]. Despite the promise of metformin, studies have yielded inconsistent results regarding its impact on sarcopenia. While some studies report a reduction in muscle mass loss in elderly T2DM patients treated with metformin,others have shown conflicting results, including reduced grip strength in some cohorts [17–19]. These inconsistencies may be attributed to small sample sizes, short durations of intervention, and unmeasured confounders, making it difficult to draw definitive conclusions.

Given the mixed results and methodological limitations (small sample sizes, short intervention periods, unmeasured confounders), the role of metformin in modulating sarcopenia risk in elderly T2DM patients remains unclear. To address this gap, our study aims to investigate the therapeutic effects of metformin on sarcopenia, focusing on key indicators such as muscle strength, lean body mass, physical function (walking pace), and related adverse events, including osteoporosis and mortality. We utilized data from the comprehensive and large-scale National Health and Nutrition Examination Survey (NHANES) to explore the relationship between metformin therapy and sarcopenia risk. In addition, we employed Mendelian randomization (MR) analysis to further investigate the potential causal relationship between metformin and sarcopenia, with the goal of providing preliminary insights into the mechanisms that may underlie its effects.

## Materials and methods

### Research design

A thorough examination of the relationship between metformin and sarcopenia has been undertaken. Initially, a cross-sectional observational analysis was conducted to assess the association between metformin usage and sarcopenia risk among T2DM patients aged over 60 years based on NHANES. Subsequently, drug target MR was employed to investigate the causal impact of metformin on various indicators related to sarcopenia. To accurately represent metformin's drug effect, cis-expression quantitative trait loci (eQTL) of downstream targets were utilized, providing insights into metformin's targeted genes that contribute to sarcopenia [20]. Furthermore, the genetic mechanisms underlying impact of metformin on sarcopenia traits were delved into by colocalization analyses. Finally, the interaction between metformin and significant targets was analyzed using molecular docking technology to ensure the robustness of the binding energy. This comprehensive approach sheds light on the multifaceted relationship between metformin and sarcopenia by integrating epidemiological, genetic, and pharmacological perspectives. Ethical approval was not required for this study as all data sources relied upon publicly available datasets. Approval for the studies included in these datasets was obtained from the relevant institutional review boards.

### Data sources

NHANES stands as a cornerstone of population-based cross-sectional research, meticulously crafted to gather insights into the health and nutritional status of individuals across various age groups in the United States. In genetic research, the Genome-Wide Association Study (GWAS) Catalog is a pivotal repository housing gene-phenotype associations data derived from an extensive published studies. The FinnGen Project and the UK Biobank are large-scale biomedical database to help us investigate the correlation between genomic information and health characteristics within a specific population. All of the analyses were implemented by R 4.3.3.

### Screening process

A total of 115,674 cases were obtained from NHANES database from 1999 to 2018 years. Study population was made up of diagnosed T2DM patients aged over 60 years. Exclusion criteria included: 1. lack of medication information and incomplete data; 2. digestive and absorption disorders caused by chronic gastrointestinal diseases; 3. depression, Parkinson's disease, dementia, cerebral infarction sequelae, cerebral hemorrhage; 4. Severe cardiopulmonary renal insufficiency; 5. various cancer; 6. administration of anti-osteoporosis, sex hormones, glucocorticoids, thyroid hormones, and antidepressants. In addition, five drug targets of metformin were identified by literature searches: PSENEN, ETFDH, GDF15, PRKAB1 and GPD1. Also, GWAS and eQTL data were downloaded at https://www.eqtlgen.org/ [21].

### Study variables and outcomes

Demographic information, including age, sex, race, and education level, as well as diabetes-related data, metformin usage, and other medication history, were gathered through baseline questionnaires. Additional data on smoking status and alcohol consumption were provided by baseline interviews, quantified using the "NHANESR" package. Participants were categorized into two groups based on their metformin usage status at baseline. Outcomes are sarcopenia-related indicators encompassing muscle strength assessed via grip strength, muscle mass assessed via Dual-Energy X-ray (DXA), lean body mass assessed and fat-free mass via bioelectrical impedance (BIA), walk pace, and incidents of osteoporosis and fragility-related events and death.

### Observational analysis

The linear relationships between metformin usage and sarcopenia-related indicators were analyzed by univariate and multivariate analyses. Multivariate linear regression was employed to adjust for various confounding factors including age, sex, race/ethnicity, height, weight, smoking status, education level, and other et.al. We also employed scatter plots to visually examine the association between duration of metformin and outcomes. Furthermore, survival analysis using the Cox regression model was conducted to explore survival differences between metformin group and non-metformin group.

### MR and eQTL colocalization analysis

Firstly, exposure and outcome variables were investigated using GWAS data,FinnGen Project and the UK Biobank. Secondly, instrumental variables strongly associated with exposure factors were selected, with a filtering condition set at a p-value < 5e-08. Then, MR analysis was conducted and the results were visually displayed. Heterogeneity was assessed using the MR-Egger tests, with p-value < 0.05 indicating the presence of heterogeneity. Once a causal link between exposure and outcome is established, and a significant signal site is identified, it is imperative to elucidate the mechanism by which the site affects these two phenotypes. Colocalization analysis is frequently utilized to determine whether two phenotypes are driven by the same causal variant in a specific genomic region, thereby strengthening the evidence of association [22]. Four hypotheses are typically considered in colocalization analysis, with the Bayesian method employed to calculate the posterior probability (PPH) values of these hypotheses. A standard criterion for screening shared SNP sites is that the PPH4 value > 0.8. In addition, this study follows the protocol of the STROBE-MR statement and provides the checklist.

### Heterogeneity and sensitivity analysis

Heterogeneity analysis addresses the potential variability of instrumental variables stemming from diverse analysis platforms, experiments, populations, etc., thereby potentially influencing the outcomes of MR. The validation of the Inverse-Variance Weighted (IVW) algorithm involved various algorithms such as weighted median method. Leave-one sensitivity analysis aimed to assess the influence of each SNP on MR analysis outcomes. If outliers were found, they were systematically removed and the analysis was re-conducted. Additionally, pleiotropic analysis was conducted to ascertain whether the instrumental variable impacted the outcome through factors beyond the exposure variable. This challenges the assumptions of independence and exclusivity. MR-Egger intercept test was utilized to detect pleiotropy and evaluate the robustness of results, with p < 0.05 indicating the presence of pleiotropy [23].

### Molecular docking

To evaluate the binding energy and interaction patterns between small molecules and their targets, a computerized protein–ligand docking software AutodockVina 1.2.2, was utilized in this study [24]. The molecular structure of metformin was obtained from the PubChem compound database, while the 3D coordinates of the protein GDF15 were downloaded from PDB (http://www.rcsb.org/) [25]. The protein and ligand files were first prepared, with all protein and molecular files converted to PDBQT format. Subsequently, water molecules were removed, and polar hydrogen atoms were added. Molecular docking studies were conducted for model visualization using Discovery Studio 4.5 software. For the same target, when the binding energy is negative, the greater the absolute value of the binding energy, the better. A binding energy of < −4.25 kcal/mol indicates a certain binding activity between the ligand small molecule and the receptor protein, while a binding energy of < − 5.0 kcal/mol suggests good binding activity between them.

## Results

**Fig. 1:**
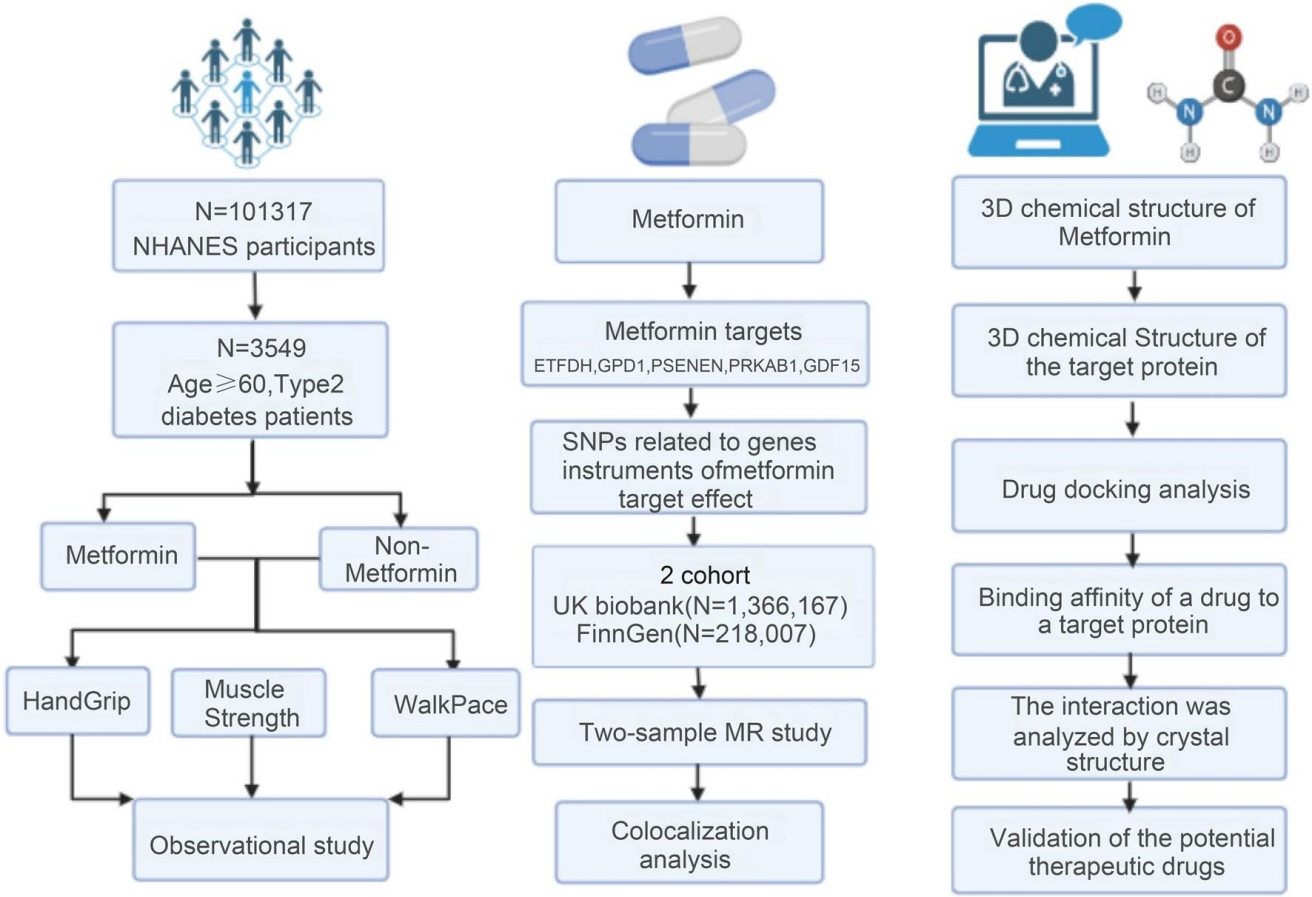
Overall design of the study

The overall idea of the paper is depicted in Fig. 1 and consists of three parts: an observational study on the association of metformin use and sarcopenia risk, MR analysis of metformin targets on sarcopenia-related traits, and molecular docking analysis of metformin targets that significantly affect sarcopenia risk.

### Observational analysis of NHANES data

#### Screening Process and baseline information

**Fig. 2:**
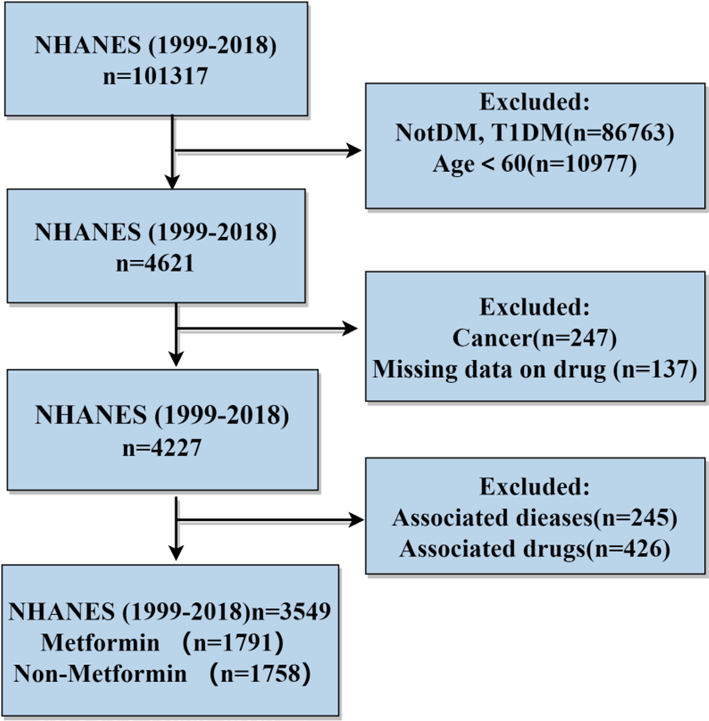
Screening process for patients in NHANES database

The flow chart of screening process is depicted in Fig. 2. A total of 4227 patients with drug use information and sarcopenia-related data were screened from 101,317 cases in NHANES (1999–2018). According to exclusion criteria, patients with associated drugs (N = 426), including osteoporosis (N = 253), using hormone drugs (N = 2), using antipsychotics (N = 171), were excluded.Patients with associated dieases(N = 245),including cardiovascular disease (N = 233), and Parkinson's disease (N = 12) were excluded. In addition, patients lack of full survival information (N = 7) were also excluded. Finally, a total of 3549 T2DM patients were enrolled, with 1791 (50.4%) receiving metformin treatment. The basic characteristics of the patients are outlined in Table S1.

### A potential reduced risk of sarcopenia was observed in the metformin group

There are differences in grip strength (p = 0.04), muscle mass (p = 0.02), walk pace (p = 0.02), bone mineral density (p = 0.01), osteoporosis and brittle (p = 0.02), and all-cause deaths (p < 0.001) between metformin and non-metformin group, as shown in Supplementary Table 2. Patients with metformin had higher grip strength [OR = 2.46, 95%CI (0.16,4.75), p = 0.04] and higher muscle mass [OR = 1.24, 95%CI (0.20,2.28), p = 0.02] than controls. 1421 deaths occurred over a median follow-up period (IQR, 11.9–13.6 years). Metformin usage was significantly negatively associated with the risk of death [HR = 0.62, 95%CI (0.54,0.71), p < 0.0001]. Metformin usage was found to be significantly associated with a reduced risk of death [HR = 0.62, 95%CI (0.54, 0.71), p < 0.0001], suggesting a potential protective effect.

### The model adjusted for multiple regression was robust

**Fig. 3:**
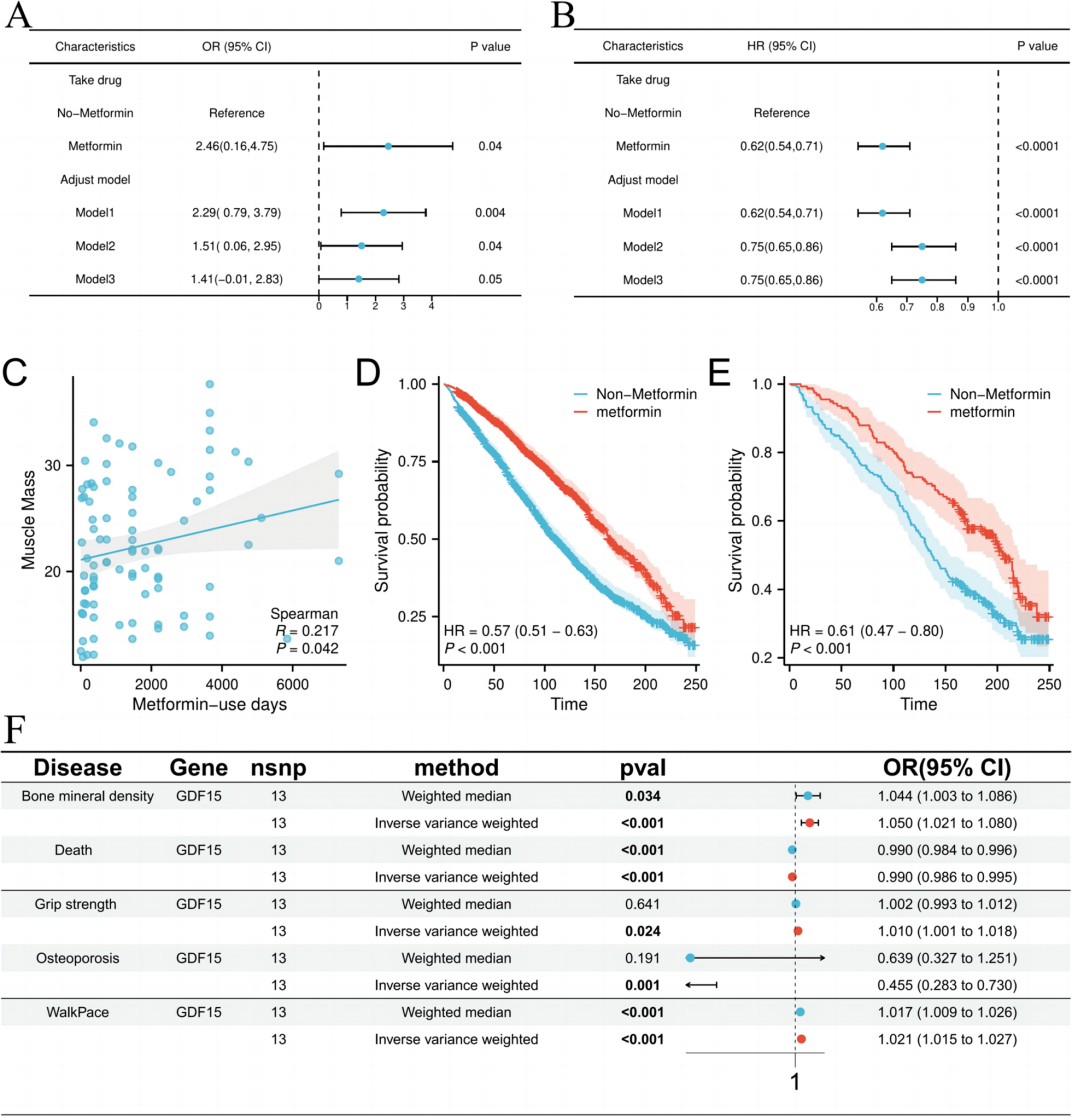
Main results from the NHANES and Drug-Targeted mendelian randomization. **A** Multifactorial linear regression between metformin use and grip strength. **B** COX survival analysis of metformin use and all-cause death. **C** Scatter plot of linear relationship between metformin use days and main study indicators. **D** Survival analysis of patients with diabetes using and without metformin for all patients included. **E** Survival analysis of patients with diabetes using and without metformin in a non-sarcopenia population. **F** Major results of Mendelian randomization of metformin drug targets for sarcopenia and adverse events

In original model, grip strength was observed to increase by 146% in patients using metformin compared to those who did not (OR = 2.46, 95% CI 1.49–2.38, p = 0.04) (Fig. 3A). This association was also evident in Model 1 after adjustment for sex (OR = 1.51; 95% CI 0.06–2.95, p = 0.004). Furthermore, this association persisted in Model 2 after adjusting for age, sex, BMI and race (OR = 2.29; 95% CI 0.79–3.79, p = 0.04). Model 3 is adjusted for smoking, there was a trend toward increased grip strength in metformin group (OR = 1.41, 95% CI 0.01—2.83, p = 0.05) (Table 1). In survival analysis, after adjusting for age in Model 1, patients using metformin exhibited a 38% lower risk of death than those not (HR = 0.62, 95% CI 0.54–0.71, p < 0.0001). This association remained significant even after full adjustment for age, sex, BMI, ethnicity, and smoking in Model 3 (HR = 0.75; 95% CI 0.65–0.86, p < 0.0001) (Fig. 3B).

**Table 1:** Multivariate regression models were used to investigate the association between metformin use and the outcome of the primary study

Varible	Crude model		Model 1			Model 2		Model 3	
	β (95% CI)	P	β (95% CI)		P	β (95% CI)	P	β(95% CI)	P
Grip strength	2.46(0.16,4.75)	0.04	2.29(0.79, 3.79)	0.004		1.51( 0.06, 2.95)	0.04	1.41(− 0.01, 2.83)	0.05
Low muscle strength	0.50(0.28,0.92)	0.03	0.50(0.27,0.94)	0.03		0.70(0.31,1.57)	0.37	0.71(0.31,1.63)	0.40
Muscle mass	1.24(0.20,2.28)	0.02	0.75(−0.10,1.61)	0.08		− 0.03(− 0.55, 0.50)	0.92	0.05(− 0.47, 0.57)	0.86
Status	0.62(0.54,0.71)	< 0.0001	0.62(0.54,0.71)	< 0.0001		0.75(0.65,0.86)	< 0.0001	0.75(0.65,0.86)	< 0.0001

OR,odds ratio, HR,hazard ratio; 95%CI,95%Confidence interval

crudel model: Metformin

model 1: Metformin + sex

model 2: Metformin + sex + age + BMI + race

model 3: Metformin + sex + age + BMI + race + smoke

### Potential association between metformin use and sarcopenia risk

A scatter plot analysis was conducted to explore the correlation between days of medication and muscle mass, and revealed a positive correlation (r = 0.217, p < 0.05) (Fig. 3C). Furthermore, Kaplan–Meier (K-M) analysis demonstrated that patients using metformin had better survival rates than those not (HR = 0.58, 95% CI 0.51–0.63, p < 0.001) (Fig. 3D). Subgroup analysis indicated similar results in non-sarcopenia patients (HR = 0.61, 95% CI 0.47–0.80, p < 0.001) (Fig. 3E). Consistent with previous results, these findings suggest that metformin may be significant value in preventing sarcopenia in T2DM patients.

### Mendelian randomization analysis

#### Genetic agent of metformin

Five downstream target genes of metformin, PSENEN, ETFDH, GDF15, PRKAB1, and GPD1 were selected as instrumental variables for metformin. Drug target Mendelian found that increased GDF15 was associated with a reduced risk of sarcopenia, as well as positively associated with a decrease in adverse events, which was the most significant effect (Table 4).

#### Potential relationship between GDF15 and sarcopenia risk, and its association with adverse events

Using samples from UK Biobank or FinnGen cohort, the effect of metformin targets on sarcopenia-related traits was investigated (Fig. 3F)(Figure S1-S7). The result revealed that GDF15 expression was positively correlated with grip strength by using the IVW as gold standard, indicating a potential causal effect (OR = 1.010, 95% CI 1.001–1.013, p = 0.024). Additionally, high expression of GDF15 was associated with high bone mineral density (OR = 1.050, 95% CI 1.021–1.080, p < 0.001), as well as increased walk speed (OR = 1.021, 95% CI 1.015–1.027, p < 0.001). Furthermore, GDF15 was found to be negatively correlated with osteoporosis (OR = 0.455, 95%CI 0.283–0.730, p = 0.001), as well as death (HR = 0.990, 95% CI 0.986–0.995, p < 0.001). While the correlation between GDF15 and lean body mass and fat-free mass did not reach statistical significance, there was a positive trend observed (Figure S2-S3) (Table 2), suggesting that GDF15, as a target of metformin, plays a significant role in reducing sarcopenia traits and associated risks.

**Table 2:** Summary of causal relationships between drug targets of metformin and major traits of sarcopenia by Mendelian randomization

Drug target	Trait	OR/HR (95% CI)	P value	GWAS id	Sample size	Number of genetic instruments	Analysis of heterogeneity	The pleiotropy analysis
GDF15	Grip strength	1.010 (1.001–1.013)	0.024	ukb-b-10215	461,089	13	0.1529	0.3743
GDF15	Lean mass	1.000 (0.985–1.014)	0.952	ebi-a-GCST90000026	205,513	13	0.3409	0.0962
GDF15	Fat free mass	1.039 (0.984–1.039)	0.163	ukb-e-23117_CSA	8,658	11	0.8639	0.2875
GDF15	Walk pace	1.021 (1.015–1.027)	< 0.001	ukb-b-4711	459,915	13	0.5175	0.7857
GDF15	Bone mineral density	1.05 (1.021–1.080)	< 0.001	ebi-a-GCST005348	56,284	13	0.6189	0.0937
GDF15	Osteoporosis	0.455 (0.283–0.730)	0.001	finn-b-OSTPOPATFRACTURE	218,163	13	0.5547	0.3296
GDF15	Death	0.990 (0.986–0.995)	< 0.001	ukb-d-ICDMAIN_ANY_ENTRY	431,365	12	0.8426	0.7140

OR,odds ratio; HR,hazard ratio; GWAS, genome-wide association study; 95%CI, 95%Confidence interval

### Heterogeneity, sensitivity, and pleiotropy analysis results were robust

Heterogeneity tests showed that there were no heterogeneity among these seven traits in Table 2. MR-Egger intercept term was utilized for the pleiotropy test, indicating the absence of horizontal pleiotropy (Table 2). A leave-one-out sensitivity analysis validated the robustness of the outcome. (Table 2). MR analysis indicated that increased GDF15 levels were associated with a positive causal effect on increased grip strength, bone mineral density, and walke pace. Additionally, elevated levels of GDF15 were linked to a detrimental impact on osteoporosis and death, indicating a beneficial feedback loop involving the metformin target GDF15 in muscle composition, skeletal muscle metabolism.

### Colocalization analysis revealed that GDF 15 shares a common genetic basis with sarcopenia

Colocalization analysis revealed that eQTL of GDF15 and grip strength were influenced by same causal mutation site. The existence of this SNP site rs4808795 was also confirmed by the posterior probability, with PPH4 value reaching 99.9%, thereby strengthening the evidence of association between two phenotypes (Figure S8). Similarly, eQTL of GDF15 shared the SNP site rs4808795 with walk pace, with the PPH4 value reaching 99.9% (Figure S9). Additionally, the SNP site rs1059369 was shared with the Bone mineral density, with the PPH4 value reaching 99.4% (Figure S10). The SNP site rs1059369 was shared with osteoporosis, with the PPH4 value reaching 97.8% (Figure S11). The same trend was observed with death, sharing the SNP site rs4808795, with the PPH4 value reaching 99.9% (Table S3) (Figure S12).

### Molecular docking showed a high affinity between metformin and GDF15

To evaluate the affinity of drug candidates to their targets, molecular docking analyses were conducted. The three-dimensional chemical structure of metformin was obtained from PubChem database for drug docking analysis, while the structure of GDF15 protein was retrieved from PCSB PDB database. To study the binding mechanism of small molecule metformin and GDF15 protein, the Discovery Studio tool was utilized to simulate molecular docking. Binding postures and interactions between drug candidates and proteins were obtained using Autodock Vina v.1.2.2, and binding energies were generated for each interaction (Figure S13A). The lowest binding energy between GDF15 and metformin was −5.541 kcal/mol, and the highest binding energy was − 4.796 kcal/mol. The results indicated that the ligand small molecules exhibited good binding activity with the receptor protein, and each drug candidate was bound to its protein target through visible hydrogen bonding and strong electrostatic interactions (Figure S13B). These interactions, as revealed by crystal structure analysis, are critical for the stable binding of metformin to GDF15. Additionally, in the two-dimensional diagram, metformin formed two hydrogen bonds with the 187th GLN amino acid glutamine residue (Figure S13C). This suggests that metformin exhibits a strong affinity for GDF15.

## Discussion

Epidemiological observations from NHANES database revealed a significant association between biguanides and sarcopenia risk. A higher association was also observed in metformin and sarcopenia-related adverse events. Since cross-sectional surveys can only observe associations, MR analysis of drug targets is utilized to provide stronger evidence. The results identified the regulation of the downstream target of metformin, GDF15, for sarcopenia traits and adverse events. Colocalization analysis confirmed that GDF15 shared a common SNP for the risk of sarcopenia traits, which further illuminates possible targets and biological mechanisms for metformin to trigger sarcopenia risk. This study utilized multiple data sources and conducted various sensitivity analyses, which suggest a potential beneficial association between metformin use and sarcopenia. Additionally, the molecular mode of action and affinity of binding metformin to GDF15 were analyzed by using molecular docking methods of network pharmacology.

The relationship between metformin and the risk of sarcopenia, such as lean body mass and grip strength, has been controversial. Some studies suggested that metformin may have potentially beneficial effects on grip strength, lean mass, walking speed, and skeletal muscle metabolism. An 18-month randomized placebo-controlled trial in Copenhagen revealed that metformin treatment benefited bone mineral content (BMC) and density (BMD) [26]. Similarly, a Chinese randomized controlled study found slight increases in trunk lean body mass and total lean body mass after 24 weeks of metformin monotherapy among type 2 diabetes patients [27]. An observational study in Japanese type 2 diabetes patients indicated that improved HbA1c levels during oral diabetes medication or insulin therapy correlated with increased walk pace [28]. In contrast, previous clinical studies indicated no significant association between metformin and sarcopenia traits. A research suggested that metformin use failed to produce significant changes in grip strength and lumbar bone density over the 24-week study period [29]. Additionally, several metformin-based combination studies investigating the effects of the drug on body composition measured by dual-energy X-rays in patients with T2DM have shown that metformin has no significant effect on lean body mass and muscle mass [30, 31]. Therefore, further studies are needed to clarify these conflicting findings and better understand the potential role of metformin in sarcopenia.

Our study observed an association between metformin use and sarcopenia-related risk in T2DM, which remained noteworthy even after adjusting for comorbidities such as smoking, alcohol consumption, education, and lifestyle factors. Previous research suggests that metformin may exert beneficial effects on age-related pathophysiology, particularly mechanisms related to energy utilization, which may impact skeletal muscle function [32]. Notably, a study investigating muscle muscle morphology in individuals with type 2 diabetes demonstrated that metformin significantly reduced muscle lipid content, potentially enhancing glucose disposal capacity in muscle muscle [33]. This mechanism may contribute to metformin's effectiveness in preventing sarcopenia in T2DM patients.

The causal relationship between metformin use and sarcopenia has not yet been explored, we therefore conducted drug-targeted MR Analyses. The results suggested a potential negative association between high GDF15 expression and sarcopenia traits, as well as adverse events such as osteoporosis and death. Furthermore, colocalization analysis identified a significant shared SNP site between eQTL expression of the GDF15 gene and the risk of sarcopenia, with a high Bayesian posterior probability verification of 99.9%, indicating a common genetic basis. Consistent with these findings, molecular docking analysis demonstrated robust interaction modes and binding energies between metformin and GDF15, suggesting a strong affinity between them. These findings collectively suggest an association between metformin use and a potentially reduced risk of sarcopenia.Mechanistically, GDF15 may play an important role in metformin regulating the progression of sarcopenia.

GDF15 is a stress response cytokine that is elevated in various chronic diseases such as cancer cachexia and chronic heart failure, and GDF15 levels are positively correlated with inflammatory markers [34]. Studies have also shown that GDF15 is negatively associated with muscle strength and lean body mass, suggesting that reducing GDF15 can mitigate these adverse effects [35]. These suggest that GDF15 affects energy metabolism and muscle function. In addition, GDF15 also plays a role in metabolic adaptation to systemic inflammation and is causally associated with T2DM, making it a therapeutic target for metabolic diseases[36]. Metformin is a common treatment for T2DM and has been shown to reduce GDF15 levels [37]. Current evidence highlights GDF15 as a key factor in sarcopenia, suggesting that metformin's effect on sarcopenia may be mediated through its effect on GDF15. While the direct molecular target of metformin remains undetermined, its effects on the lysosomal AMPK pathway and subsequent effects on GDF15 highlight its potential role in improving muscle health and reducing the risk of sarcopenia [38]. Further exploration of the mechanisms by which metformin affects GDF15 and sarcopenia is needed to confirm these findings and develop targeted therapies.

This study have several strengths. First,the Mendelian randomization (MR) analysis utilized high-quality GWAS data to establish causal relationships between metformin use and sarcopenia-related traits. Second, the colocalization analysis identified a shared causal variant between metformin and sarcopenia, strengthening the evidence for a mechanistic role of metformin in modulating sarcopenia risk. Third, the NHANES dataset was meticulously curated with strict inclusion and exclusion criteria, minimizing the influence of other medications on the primary study outcomes. Despite these strengths, several limitations should be considered. First, while MR analysis provides causal insights, it may be influenced by weak instrument bias or pleiotropy, which could undermine the magnitude of the causal estimate. We have discussed the potential direction and magnitude of these biases, acknowledging that they likely lead to more conservative estimates of the true effect of metformin on sarcopenia. Another limitation of this study is the lack of data on metformin dosage in the NHANES database, which precluded a direct analysis of dose–response relationships. However, we utilized the available data on the duration of metformin use to perform a scatterplot analysis of its correlation with muscle mass, a key indicator of sarcopenia. This analysis provided preliminary validation and revealed a modest positive association, suggesting that longer durations of metformin use may be linked to higher muscle mass. Future studies with more granular clinical data, including detailed metformin dosage information, are needed to further explore and validate these findings.Furthermore, it is important to note that this study is observational in nature, and as such, causality cannot be definitively concluded. While our findings suggest an association between metformin use and sarcopenia risk reduction, further randomized controlled trials (RCTs) are required to validate these associations and establish causal relationships.

## Conclusion

In conclusion, our findings suggest that metformin may have a beneficial impact on muscle strength and mass in elderly patients with type 2 diabetes, potentially reducing the risk of sarcopenia. However, while the evidence is promising, further large-scale and multicenter studies are needed to validate these findings and fully elucidate the causal relationship between metformin use and sarcopenia risk. Additionally, our analysis points to GDF15 as a potential target for future investigations, though more research is required to confirm its role in modulating sarcopenia.

## Supplementary Information

Supplementary material 1

Supplementary material 2

Supplementary material 3

## Data Availability

The datasets underlying this article were derived from sources in the NHANES (https://www.cdc.gov/nchs/nhanes/index.htm); eQTL database (https://www.eqtlgen.org/cis-eqtls.html); GWAS Project (https://gwas.mrcieu.ac.uk/).

## Abbreviations

NHANES: National Health and Nutrition Examination Survey
MR: Mendelian randomization
GWAS: Genome-wide association study
eQTL: Expression quantitative trait Loci
OR: Odds ratio
HR: Hazard ratio
T2DM: Type 2 diabetes mellitus
DXA: Dual-Energy X-ray
BIA: Bioelectrical impedance analysis
IVW: Inverse-Variance Weighted
95%CI,95%: Confidence interval.
K–M: Kaplan–Meier
BMC: Bone mineral content
BMD: Bone mineral density

## References

[1] Campo-Rivera N, Ocampo-Chaparro JM, Carvajal-Ortiz R, Reyes-Ortiz CA. Sarcopenic dysphagia is associated with mortality in institutionalized older adults. J Am Med Dir Assoc. 2022;23(10):1720 e11-e17. 10.1016/j.jamda.2022.06.016 35868351

[2] Anagnostis P, Gkekas NK, Achilla C, Pananastasiou G, Taouxidou P, Mitsiou M, et al. Type 2 diabetes mellitus is associated with increased risk of sarcopenia: a systematic review and meta-analysis. Calcif Tissue Int. 2020;107(5):453–63. 32772138 10.1007/s00223-020-00742-y

[3] Chung SM, Moon JS, Chang MC. Prevalence of sarcopenia and its association with diabetes: a meta-analysis of community-dwelling asian population. Front Med. 2021;8: 681232. 10.3389/fmed.2021.681232 PMC8174659 34095184

[4] Lin CC, Ou HY, Hsu HY, Cheng KP, Hsieh TJ, Yeh CH, et al. Beyond Sarcopenia: older adults with type II diabetes mellitus tend to experience an elevated risk of poor dynamic balance-a case-control study. BMC Geriatr. 2022;22(1):138. 35177026 10.1186/s12877-022-02826-w PMC8855561

[5] Mesinovic J, Zengin A, De Courten B, Ebeling PR, Scott D. Sarcopenia and type 2 diabetes mellitus: a bidirectional relationship. Diabetes Metab Syndr Obes. 2019;12:1057–72. 31372016 10.2147/DMSO.S186600 PMC6630094

[6] de Luis RD, Garrachon Vallo F, Carretero Gomez J, Lopez Gomez JJ, Tarazona Santabalbina FJ, Guzman Rolo G, et al. Decreased muscle mass in type-2 diabetes. A hidden comorbidity to consider. Nutr Hosp. 2023;40(1):59–66. 36633517 10.20960/nh.04468

[7] de Luis Roman D, Gomez JC, Garcia-Almeida JM, Vallo FG, Rolo GG, Gomez JJL, et al. Diabetic Sarcopenia. A proposed muscle screening protocol in people with diabetes : Expert document. Rev Endocr Metab Disord. 2024. 10.1007/s11154-023-09871-9 PMC11294263 38315411

[8] Singh A, Buckholz A, Kumar S, Newberry C. Implications of protein and sarcopenia in the prognosis, treatment, and management of metabolic dysfunction-associated steatotic liver disease (MASLD). Nutrients. 2024;16(5):658. 38474786 10.3390/nu16050658 PMC10933907

[9] Zheng Y, Feng J, Yu Y, Ling M, Wang X. Advances in sarcopenia: mechanisms, therapeutic targets, and intervention strategies. Arch Pharm Res. 2024;47(4):301–24. 38592582 10.1007/s12272-024-01493-2

[10] Mellen RH, Girotto OS, Marques EB, Laurindo LF, Grippa PC, Mendes CG, et al. Insights into pathogenesis, nutritional and drug approach in sarcopenia: a systematic review. Biomedicines. 2023;11(1):136. 36672642 10.3390/biomedicines11010136 PMC9856128

[11] Witham MD, Granic A, Pearson E, Robinson SM, Sayer AA. Repurposing drugs for diabetes mellitus as potential pharmacological treatments for sarcopenia—a narrative review. Drugs Aging. 2023;40(8):703–19. 37486575 10.1007/s40266-023-01042-4 PMC10371965

[12] Bang S, Kim DE, Kang HT, Lee JH. Metformin restores autophagic flux and mitochondrial function in late passage myoblast to impede age-related muscle loss. Biomed Pharmacother. 2024;180: 116981. 39533541 10.1016/j.biopha.2024.116981

[13] Tezze C, Amendolagine FI, Nogara L, Baraldo M, Ciciliot S, Arcidiacono D, et al. A combination of metformin and galantamine exhibits synergistic benefits in the treatment of sarcopenia. JCI Insight. 2023. 10.1172/jci.insight.168787. 37551712 10.1172/jci.insight.168787 PMC10445681

[14] Santulli G, Visco V, Varzideh F, Guerra G, Kansakar U, Gasperi M, et al. Prediabetes increases the risk of frailty in prefrail older adults with hypertension: beneficial effects of metformin. Hypertension. 2024;81(7):1637–43. 38752357 10.1161/HYPERTENSIONAHA.124.23087 PMC11170724

[15] Mone P, Martinelli G, Lucariello A, Leo AL, Marro A, De Gennaro S, et al. Extended-release metformin improves cognitive impairment in frail older women with hypertension and diabetes: preliminary results from the LEOPARDESS study. Cardiovasc Diabetol. 2023;22(1):94. 37085892 10.1186/s12933-023-01817-4 PMC10122301

[16] Mone P, Gambardella J, Pansini A, de Donato A, Martinelli G, Boccalone E, et al. Cognitive impairment in frail hypertensive elderly patients: role of hyperglycemia. Cells. 2021;10(8):2115. 34440883 10.3390/cells10082115 PMC8391431

[17] Lyu Q, Wen Y, He B, Zhang X, Chen J, Sun Y, et al. The ameliorating effects of metformin on disarrangement ongoing in gastrocnemius muscle of sarcopenic and obese sarcopenic mice. Biochim Biophys Acta Mol Basis Dis. 2022;1868(11): 166508. 35905940 10.1016/j.bbadis.2022.166508

[18] Long DE, Kosmac K, Dungan CM, Bamman MM, Peterson CA, Kern PA. Potential benefits of combined statin and metformin therapy on resistance training response in older individuals. Front Physiol. 2022;13: 872745. 35492586 10.3389/fphys.2022.872745 PMC9047873

[19] Hernandez-Arciga U, Hernandez-Alvarez D, Lopez-Cervantes SP, Lopez-Diazguerrero NE, Alarcon-Aguilar A, Luna-Lopez A, et al. Effect of long-term moderate-exercise combined with metformin-treatment on antioxidant enzymes activity and expression in the gastrocnemius of old female Wistar rats. Biogerontology. 2020;21(6):787–805. 32749628 10.1007/s10522-020-09894-8

[20] Consortium GT. Human genomics. The genotype-tissue expression (GTEx) pilot analysis: multitissue gene regulation in humans. Science. 2015;348(6235):648–60. 25954001 10.1126/science.1262110 PMC4547484

[21] Zhu Z, Zhang F, Hu H, Bakshi A, Robinson MR, Powell JE, et al. Integration of summary data from GWAS and eQTL studies predicts complex trait gene targets. Nat Genet. 2016;48(5):481–7. 27019110 10.1038/ng.3538

[22] Zuber V, Grinberg NF, Gill D, Manipur I, Slob EAW, Patel A, et al. Combining evidence from Mendelian randomization and colocalization: review and comparison of approaches. Am J Hum Genet. 2022;109(5):767–82. 35452592 10.1016/j.ajhg.2022.04.001 PMC7612737

[23] Bowden J, Davey Smith G, Burgess S. Mendelian randomization with invalid instruments: effect estimation and bias detection through Egger regression. Int J Epidemiol. 2015;44(2):512–25. 26050253 10.1093/ije/dyv080 PMC4469799

[24] Morris GM, Huey R, Olson AJ. Using AutoDock for ligand-receptor docking. Curr Protoc Bioinformatics. 2008;Chapter 8:Unit 8 14. 10.1002/0471250953.bi0814s24 19085980

[25] Li J, Shang G, Chen YJ, Brautigam CA, Liou J, Zhang X, et al. Cryo-EM analyses reveal the common mechanism and diversification in the activation of RET by different ligands. Elife. 2019. 10.7554/eLife.47650. 31535977 10.7554/eLife.47650 PMC6760901

[26] Nordklint AK, Almdal TP, Vestergaard P, Lundby-Christensen L, Boesgaard TW, Breum L, et al. Effect of metformin and insulin vs. placebo and insulin on whole body composition in overweight patients with type 2 diabetes: a randomized placebo-controlled trial. Osteoporos Int. 2021; 32(9): 1837–48. 10.1007/s00198-021-05870-1 33594488

[27] Feng WH, Bi Y, Li P, Yin TT, Gao CX, Shen SM, et al. Effects of liraglutide, metformin and gliclazide on body composition in patients with both type 2 diabetes and non-alcoholic fatty liver disease: a randomized trial. J Diabetes Investig. 2019;10(2):399–407. 29957886 10.1111/jdi.12888 PMC6400178

[28] Sugimoto K, Ikegami H, Takata Y, Katsuya T, Fukuda M, Akasaka H, et al. Glycemic Control and Insulin Improve Muscle Mass and Gait Speed in Type 2 Diabetes: The MUSCLES-DM Study. J Am Med Dir Assoc. 2021;22(4):834-8 e1. 33278348 10.1016/j.jamda.2020.11.003

[29] Laksmi PW, Setiati S, Tamin TZ, Soewondo P, Rochmah W, Nafrialdi N, et al. Effect of metformin on handgrip strength, gait speed, myostatin serum level, and health-related quality of life: a double blind randomized controlled trial among non-diabetic pre-frail elderly patients. Acta Med Indones. 2017;49(2):118–27. 28790226

[30] Koshizaka M, Ishikawa K, Ishibashi R, Maezawa Y, Sakamoto K, Uchida D, et al. Effects of ipragliflozin versus metformin in combination with sitagliptin on bone and muscle in Japanese patients with type 2 diabetes mellitus: Subanalysis of a prospective, randomized, controlled study (PRIME-V study). J Diabetes Investig. 2021;12(2):200–6. 32623839 10.1111/jdi.13340 PMC7858125

[31] McCrimmon RJ, Catarig AM, Frias JP, Lausvig NL, le Roux CW, Thielke D, et al. Effects of once-weekly semaglutide vs once-daily canagliflozin on body composition in type 2 diabetes: a substudy of the SUSTAIN 8 randomised controlled clinical trial. Diabetologia. 2020;63(3):473–85. 31897524 10.1007/s00125-019-05065-8 PMC6997246

[32] Joseph GA, Wang SX, Jacobs CE, Zhou W, Kimble GC, Tse HW, et al. Partial inhibition of mTORC1 in aged rats counteracts the decline in muscle mass and reverses molecular signaling associated with sarcopenia. Mol Cell Biol. 2019. 10.1128/MCB.00141-19. 31308131 10.1128/MCB.00141-19 PMC6751631

[33] Foretz M, Guigas B, Viollet B. Understanding the glucoregulatory mechanisms of metformin in type 2 diabetes mellitus. Nat Rev Endocrinol. 2019;15(10):569–89. 31439934 10.1038/s41574-019-0242-2

[34] Sawalha K, Norgard NB, Drees BM, Lopez-Candales A. Growth differentiation factor 15 (GDF-15), a new biomarker in heart failure management. Curr Heart Fail Rep. 2023;20(4):287–99. 37289373 10.1007/s11897-023-00610-4

[35] Chiariello A, Conte G, Rossetti L, Trofarello L, Salvioli S, Conte M. Different roles of circulating and intramuscular GDF15 as markers of skeletal muscle health. Front Endocrinol. 2024;15:1404047. 10.3389/fendo.2024.1404047 PMC11130406 38808117

[36] Au Yeung SL, Luo S, Schooling CM. The impact of GDF-15, a biomarker for metformin, on the risk of coronary artery disease, breast and colorectal cancer, and type 2 diabetes and metabolic traits: a Mendelian randomisation study. Diabetologia. 2019;62(9):1638–46. 31161347 10.1007/s00125-019-4913-2

[37] Aguilar-Recarte D, Barroso E, Palomer X, Wahli W, Vazquez-Carrera M. Knocking on GDF15’s door for the treatment of type 2 diabetes mellitus. Trends Endocrinol Metab. 2022;33(11):741–54. 36151002 10.1016/j.tem.2022.08.004

[38] Aguilar-Recarte D, Barroso E, Zhang M, Rada P, Pizarro-Delgado J, Pena L, et al. A positive feedback loop between AMPK and GDF15 promotes metformin antidiabetic effects. Pharmacol Res. 2023;187: 106578. 36435271 10.1016/j.phrs.2022.106578

