# Supplementary material for: Exploring the protective effect of metformin against sarcopenia: insights from cohort studies and genetics
Source: J Transl Med. 2025 Mar 21;23:356. doi: 10.1186/s12967-025-06357-x (PMC11927167; doi:10.1186/s12967-025-06357-x)
Supplement: Supplementary file 1 — Supplementary material 1 [file 12967_2025_6357_MOESM1_ESM.docx]

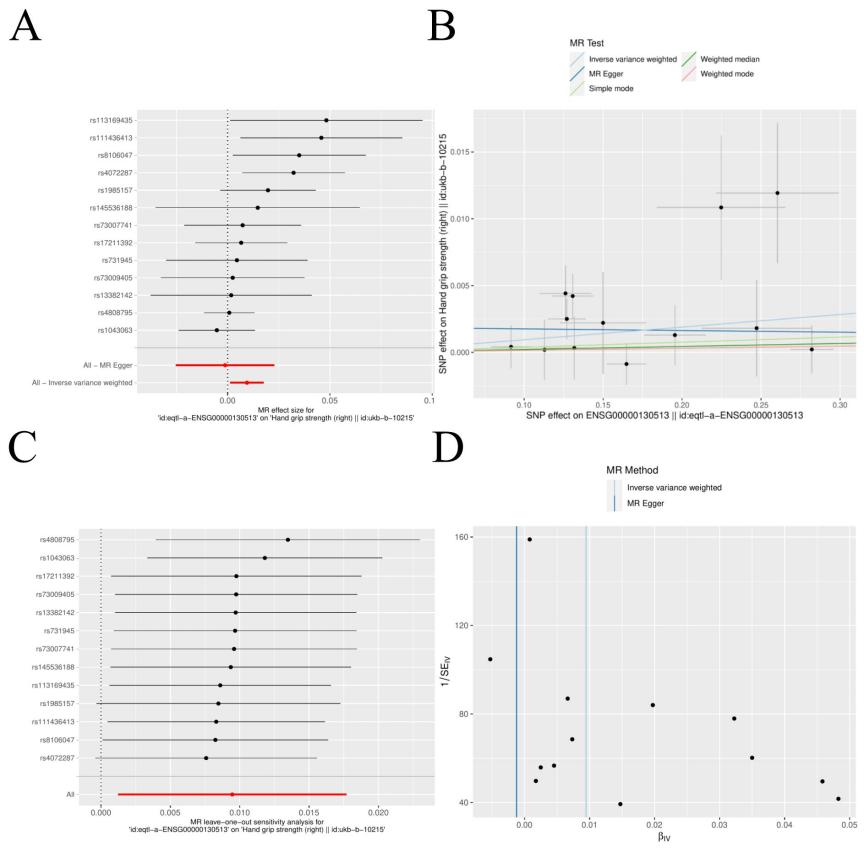


**FigureS1 .Mendelian randomization analysis of GDF15 and Hand grip strength**

(A) Forest map

(B) Scatter plot

(C) leave-one-SNP-out sensitivity analysis

(D) Funnel diagram


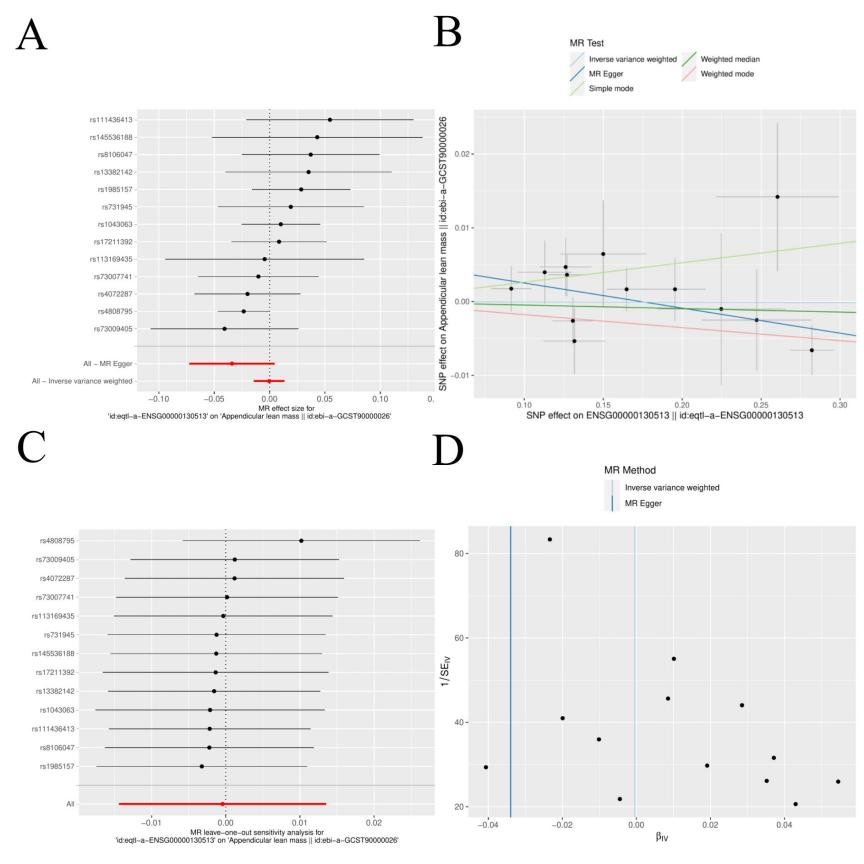


**FigureS2 .Mendelian randomization analysis of GDF15 and Appendicular lean mass.**

(A) Forest map

(B) Scatter plot

(C) leave-one-SNP-out sensitivity analysis

(D) Funnel diagram


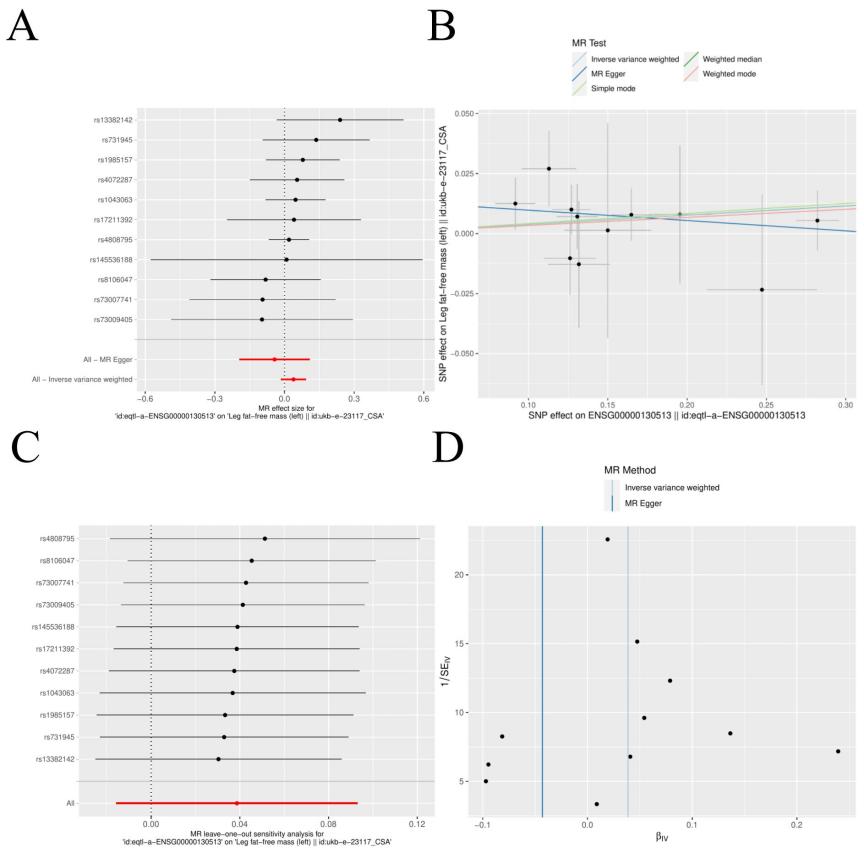


**FigureS3 .Mendelian randomization analysis of GDF15 and Leg fat−free mass (left).**

(A) Forest map

(B) Scatter plot

(C) leave-one-SNP-out sensitivity analysis

(D) Funnel diagram


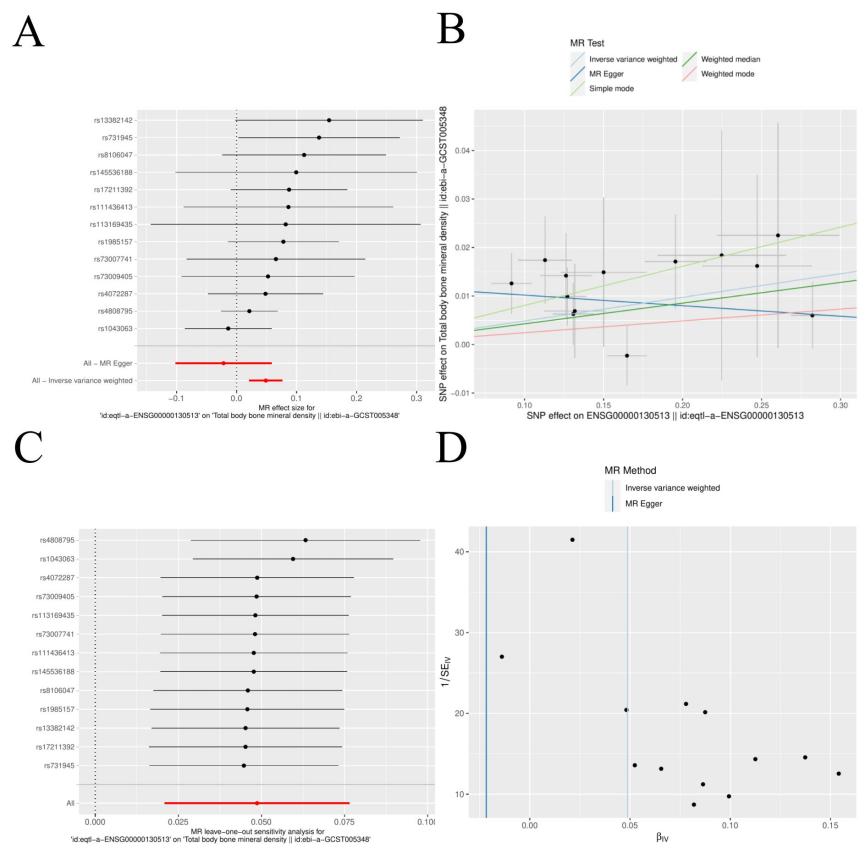


**FigureS4 .Mendelian randomization analysis of GDF15 and Total body bone mineral density.**

(A) Forest map

(B) Scatter plot

(C) leave-one-SNP-out sensitivity analysis

(D) Funnel diagram


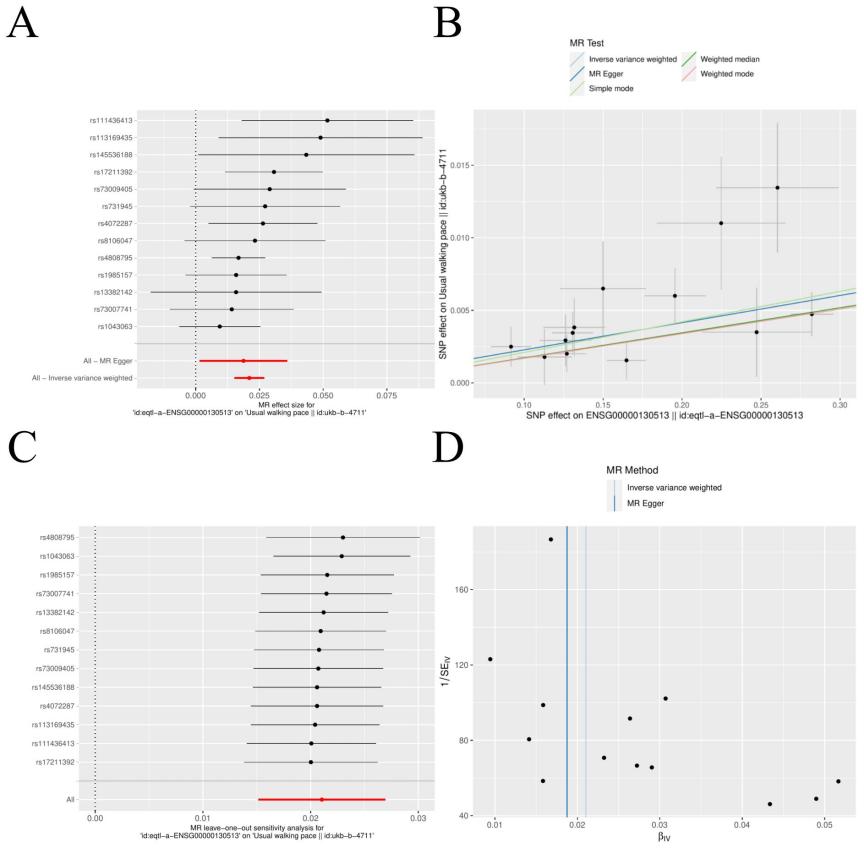


**FigureS5 .Mendelian randomization analysis of GDF15 and Usual walking pace.**

(A) Forest map

(B) Scatter plot

(C) leave-one-SNP-out sensitivity analysis

(D) Funnel diagram


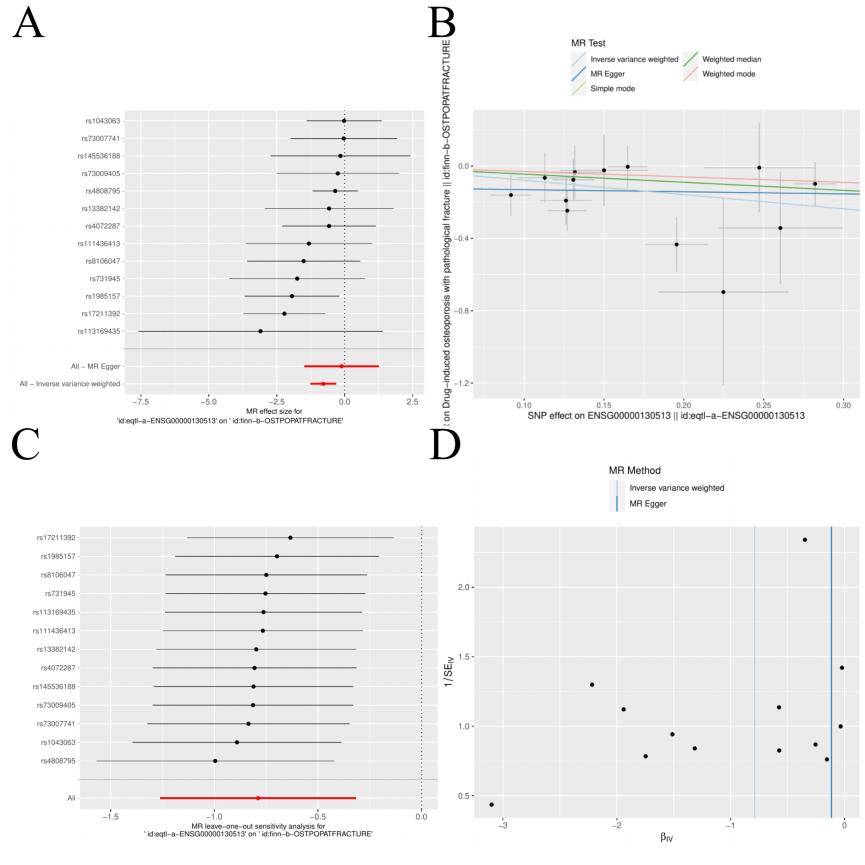


**FigureS6.Mendelian randomization analysis of GDF15 and Drug−induced osteoporosis with pathological fracture.**

(A) Forest map

(B) Scatter plot

(C) leave-one-SNP-out sensitivity analysis

(D) Funnel diagram


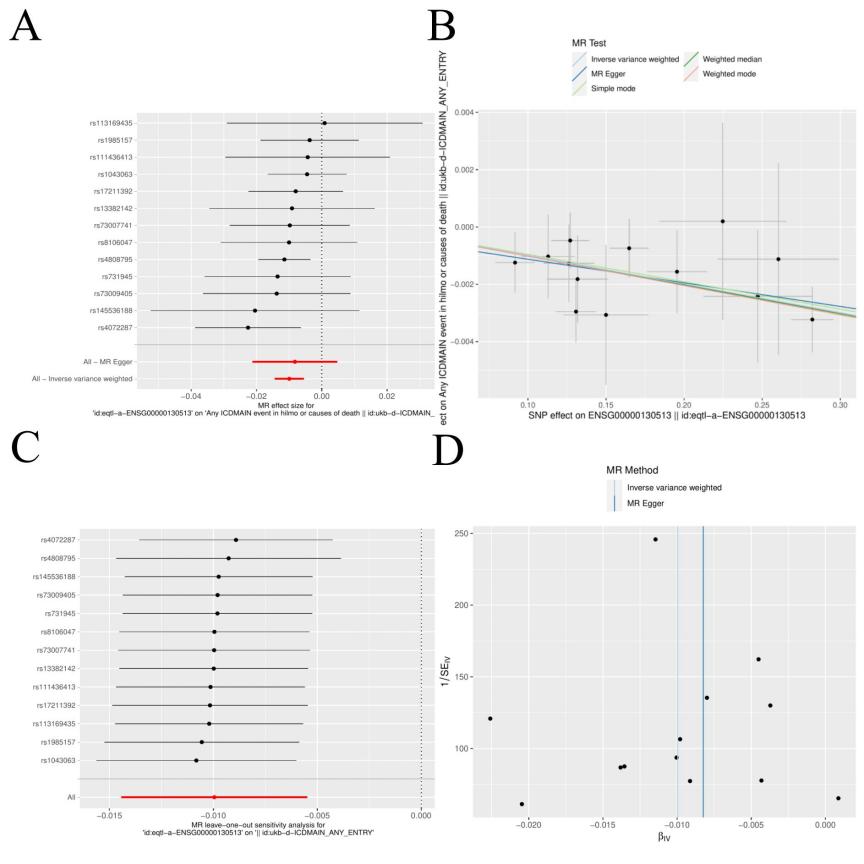


**FigureS7.Mendelian randomization analysis of GDF15 and Any ICDMAIN event in hilmo or causes of death.**

(A) Forest map

(B) Scatter plot

(C) leave-one-SNP-out sensitivity analysis

(D) Funnel diagram


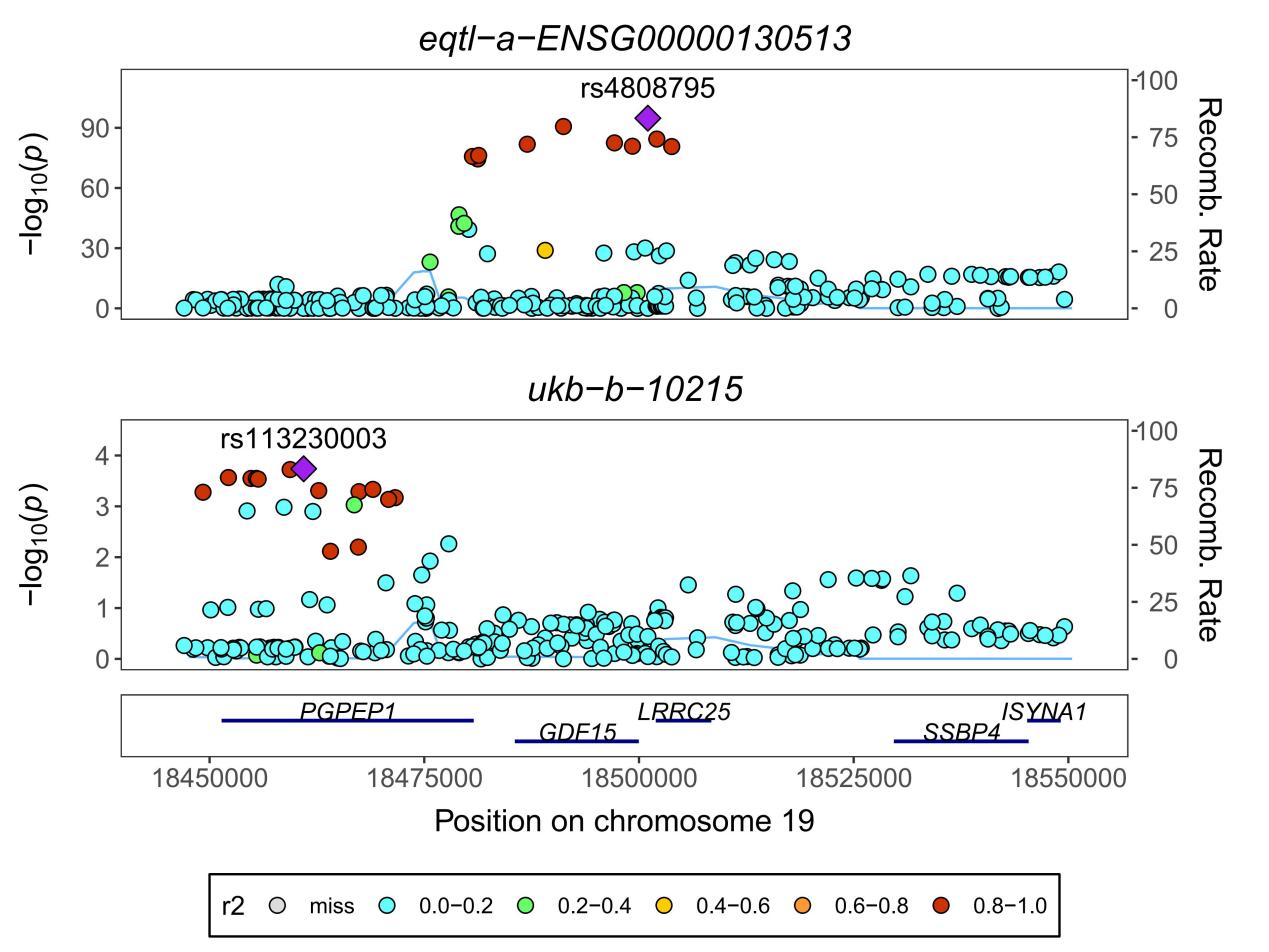


**FigureS8 . coloc co-location analysis of GDF15 and ukb-b-10215.**


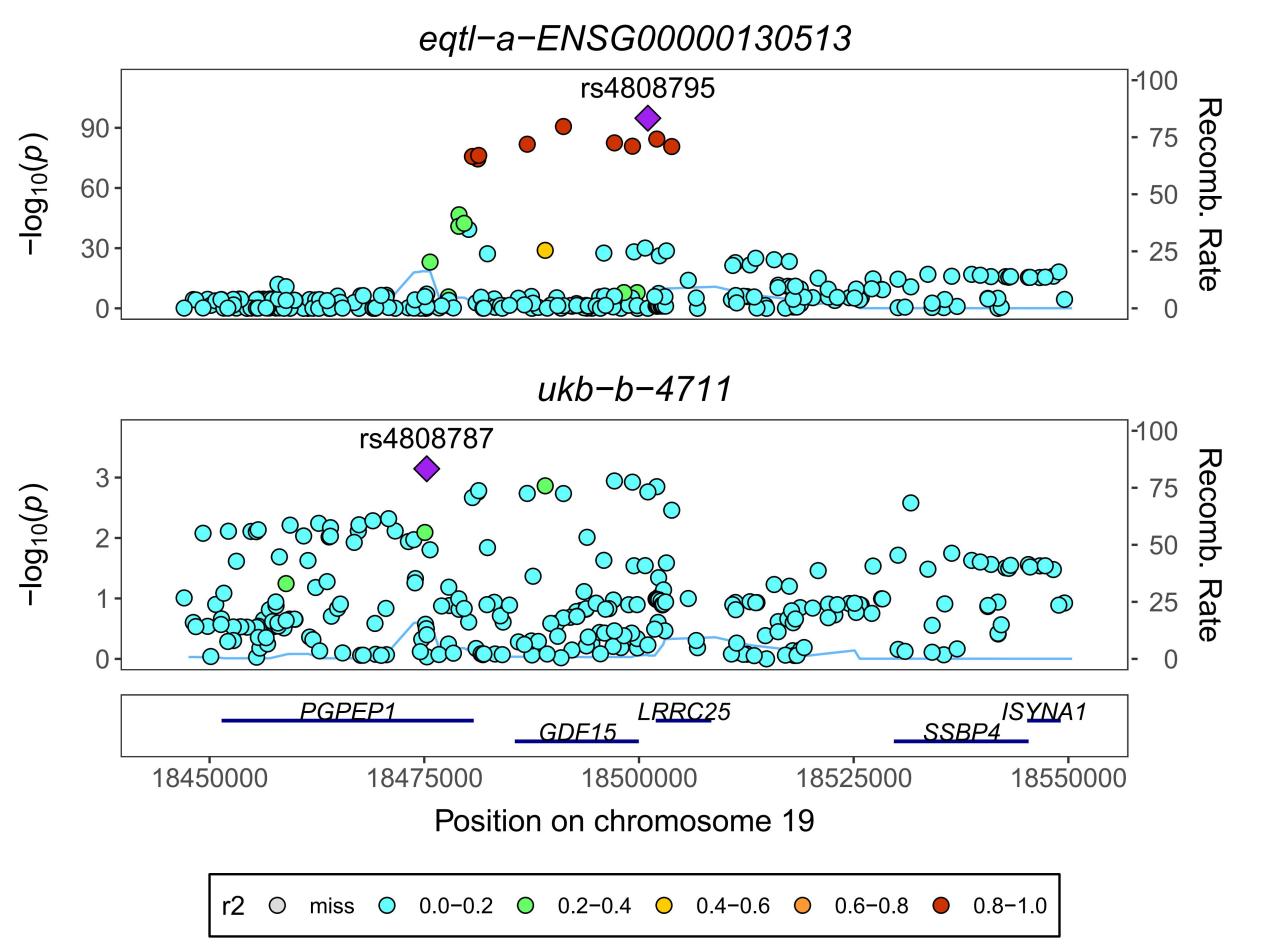
**FigureS9. coloc co-location analysis of GDF15 and ukb-b-4711.**


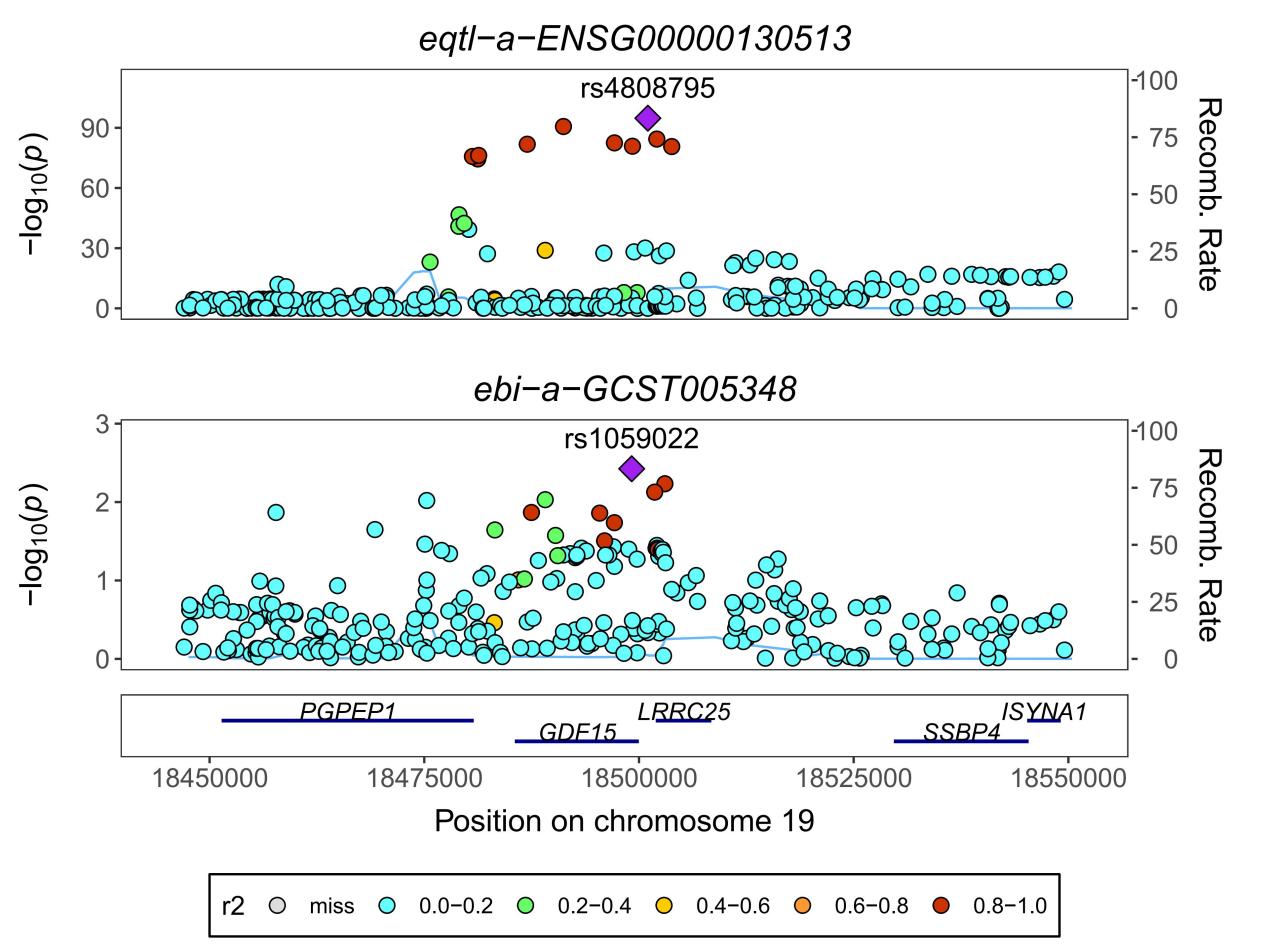
**FigureS10. coloc co-location analysis of GDF15 and ebi-a-GCST005348.**


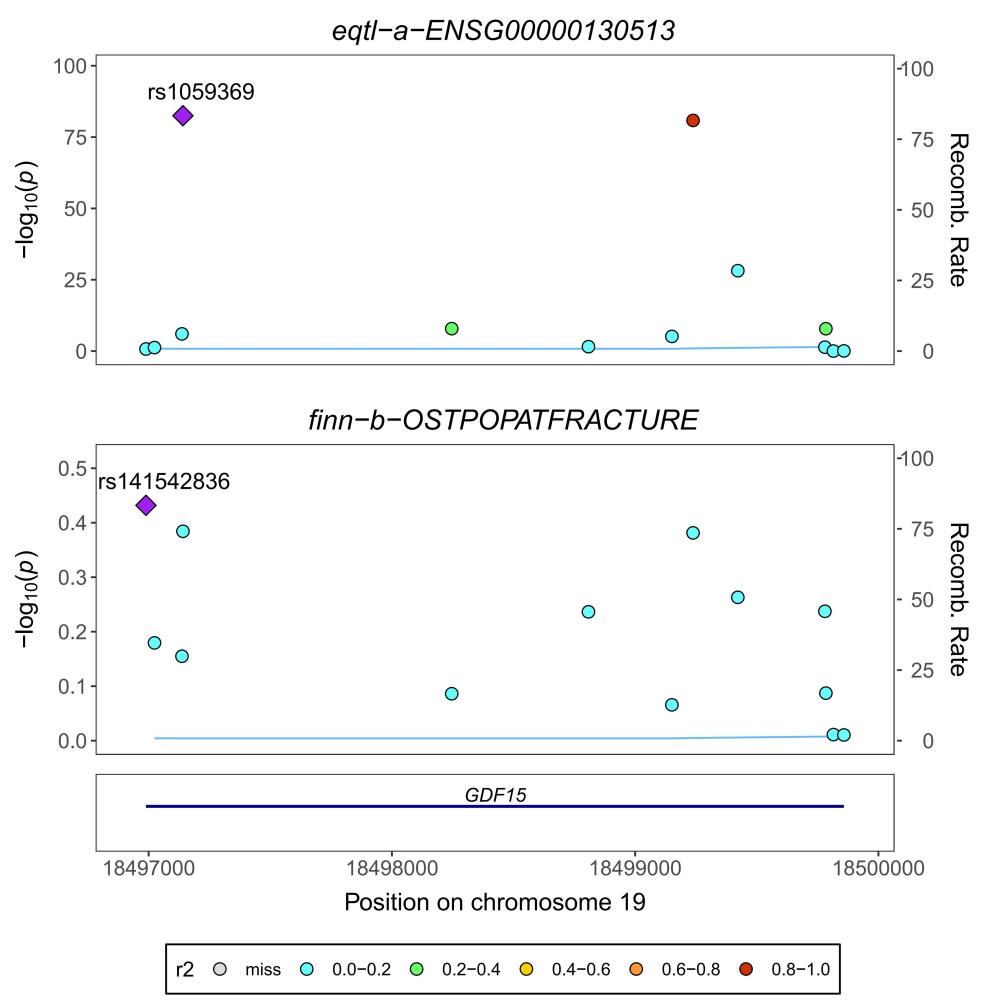


**FigureS11. coloc co-location analysis of GDF15 and finn-b-OSTPOPATFRACTURE.**


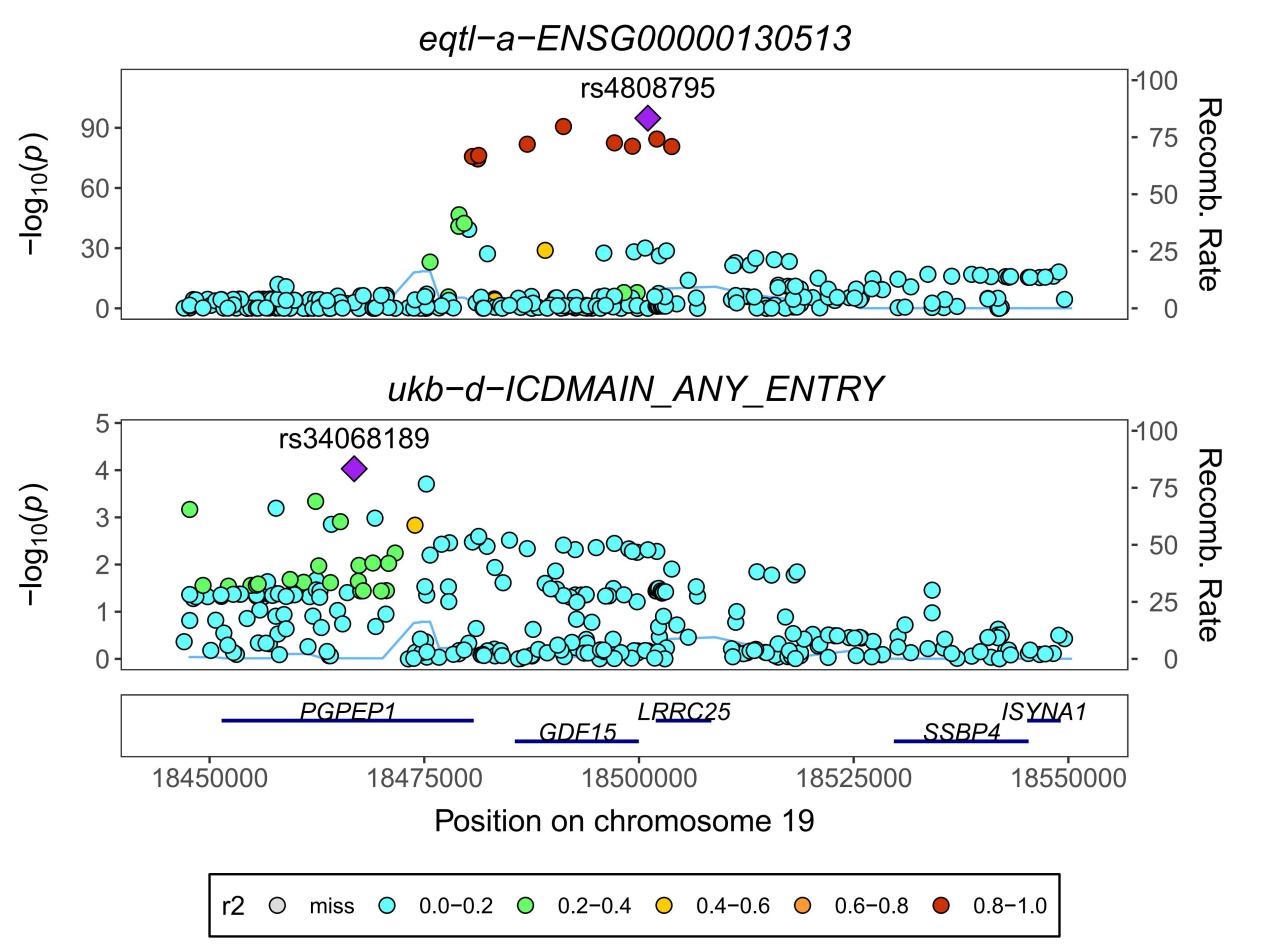


**FigureS12. coloc co-location analysis of GDF15 and ukb-d-ICDMAIN_ANY_ENTRY.**


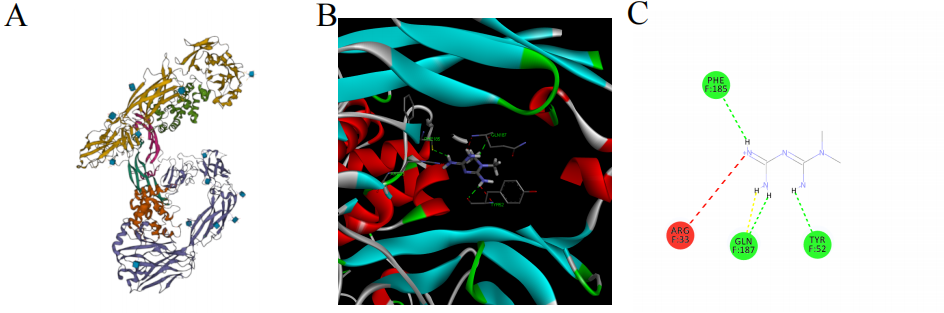


**FigureS13. Screening drugs are bound to their targets by molecular docking.**

(A) Visualization of molecular docking

(B) 3-dimensional diagram of how metformin is combined with GDF15.

(C) A 2-dimensional diagram of residual-ligand interactions.
