# Supplementary material for: Exploring the protective effect of metformin against sarcopenia: insights from cohort studies and genetics
Source: J Transl Med. 2025 Mar 21;23:356. doi: 10.1186/s12967-025-06357-x (PMC11927167; doi:10.1186/s12967-025-06357-x)
Supplement: Supplementary file 2 — Supplementary material 2 [file 12967_2025_6357_MOESM2_ESM.docx]

**Supplementary TableS1. Baseline data of T2DM patients in NHANES database.**

| Variable | N | Metformin | Non-Metformin | P |
| --- | --- | --- | --- | --- |
| Age | 70.00(65.00,76.00) | 68.00(64.00,74.00) | 71.00(65.00,78.00) | < 0.0001 |
| Weight | 84.80(73.00,99.40) | 87.00(74.30,101.10) | 83.20(71.00, 97.10) | 0.002 |
| Height | 166.10(158.70,173.60) | 166.70(159.60,174.30) | 165.30(157.60,172.90) | 0.01 |
| BMI | 30.61(26.90,35.30) | 30.98(27.38,35.30) | 30.30(26.55,35.20) | 0.07 |
| Ageclass |  |  |  | < 0.0001 |
| [60,70) | 1722(49.16) | 987(55.10) | 735(42.81) |  |
| [70,80) | 1189(34.22) | 592(33.92) | 597(34.54) |  |
| [80,90) | 638(16.62) | 212(10.98) | 426(22.65) |  |
| Smoke |  |  |  | 0.54 |
| former | 1439(42.32) | 708(41.67) | 731(43.04) |  |
| never | 1740(48.32) | 876(48.26) | 864(48.40) |  |
| now | 368( 9.34) | 207(10.07) | 161( 8.56) |  |
| Alcohol |  |  |  | < 0.0001 |
| former | 1007(25.70) | 470(28.47) | 537(32.37) |  |
| heavy | 190( 4.46) | 122(5.68) | 68(4.82) |  |
| mild | 897(31.57) | 501(42.67) | 396(31.50) |  |
| moderate | 202( 6.24) | 113(7.56) | 89(7.17) |  |
| never | 639(16.70) | 294(15.61) | 345(24.14) |  |
| Sex |  |  |  | 0.01 |
| Female | 1696(49.35) | 838(46.50) | 858(52.39) |  |
| Male | 1853(50.65) | 953(53.50) | 900(47.61) |  |
| Race |  |  |  | 0.002 |
| Mexican American | 677( 6.43) | 380(7.51) | 297(5.29) |  |
| Non-Hispanic Black | 898(13.30) | 407(11.75) | 491(14.95) |  |
| Non-Hispanic White | 1387(68.22) | 657(67.49) | 730(68.99) |  |
| Other Hispanic | 294( 4.20) | 170(4.42) | 124(3.97) |  |
| Other Race | 293( 7.85) | 177(8.83) | 116(6.80) |  |

IQR, interquartile range.

**Supplementary TableS2. Univariate analysis of metformin versus non-metformin patients in the NHANES database.**

| Variable | N | Metformin | Non-Metformin | P |
| --- | --- | --- | --- | --- |
| Grip strength | 31.62(30.22,33.02) | 32.64(30.85,34.43) | 30.18(28.43,31.94) | 0.04 |
| Grip strength max | 60.22(57.44,63.01) | 62.14(58.72,65.56) | 57.49(54.03,60.94) | 0.03 |
| Calf circumference | 38.11(37.65,38.56) | 38.66(37.95,39.37) | 37.78(37.20,38.35) | 0.06 |
| Walk pace | 7.83(7.41,8.25) | 7.31(6.78,7.84) | 8.07(7.59,8.56) | 0.02 |
| Muscle mass | 21.63(21.10,22.17) | 22.40(21.55,23.25) | 21.16(20.51,21.81) | 0.02 |
| BMC | 51.5(50.32,52.60) | 53.21(51.45,54.96) | 50.38(48.99,51.77) | 0.01 |
| BMD | 1.10(1.09,1.11) | 1.11(1.09,1.13) | 1.09(1.07,1.10) | 0.02 |
| Low muscle strength |  |  |  | 0.02 |
| No | 523(17.34) | 304(90.28) | 219(82.41) |  |
| Yes | 86( 2.59) | 40( 9.72) | 46(17.59) |  |
| Osteoporosis and brittle |  |  |  | 0.02 |
| No | 2423(65.18) | 1202(88.90) | 1221(85.10) |  |
| Yes | 309( 9.77) | 136(11.10) | 173(14.90) |  |
| Status |  |  |  | < 0.0001 |
| Alive | 2128(63.33) | 1270(72.89) | 858(53.11) |  |
| Dead | 1421(36.67) | 521(27.11) | 900(46.89) |  |

IQR, interquartile range; BMC,bone mineral content; BMD, bone mineral density;

**Supplementary TableS3. Colocalization analysis shows the posterior probability between exposure and outcome.**

| Drug target | Trait | Colocalization analysis | |
| --- | --- | --- | --- |
|  |  | PPH4 | snp |
| GDF15 | Grip strength | 99.9% | rs4808795 |
| GDF15 | Walk pace | 99.9% | rs4808795 |
| GDF15 | Bone mineral density | 99.4% | rs1059369 |
| GDF15 | Osteoporosis | 97.8% | rs1059369 |
| GDF15 | Death | 99.9% | rs4808795 |
