# Supplementary material for: Exploring the protective effect of metformin against sarcopenia: insights from cohort studies and genetics
Source: J Transl Med. 2025 Mar 21;23:356. doi: 10.1186/s12967-025-06357-x (PMC11927167; doi:10.1186/s12967-025-06357-x)
Supplement: Supplementary file 3 — Supplementary material 3 [file 12967_2025_6357_MOESM3_ESM.docx]

**STROBE-MR checklist of recommended items to address in reports of Mendelian randomization studies**^1^ ^2^

| **Item No.** | **Section** | **Checklist item** | **Page No.** | **Relevant text from manuscript** |
| --- | --- | --- | --- | --- |
| 1 | **TITLE and ABSTRACT** | Indicate Mendelian randomization (MR) as the study’s design in the title and/or the abstract if that is a main purpose of the study | 1-4 | Title:Exploring the protective effect of metformin against sarcopenia: insights from cohort studies and genetics  Abstract :Mendelian randomization (MR) analysis was also conducted to ascertain the causal relationship between drug targets and sarcopenia traits, utilizing genome-wide association study (GWAS) data from UK Biobank (n = 1,366,167) and FinnGen (n = 218,007). |
|  | **INTRODUCTION** |  | 5 |  |
| 2 | **Background** | Explain the scientific background and rationale for the reported study. What is the exposure? Is a potential causal relationship between exposure and outcome plausible? Justify why MR is a helpful method to address the study question | 5-6 | By MR analysis, we aimed to evaluate more accurately the potential mechanism of action and clinical efficacy of metformin on sarcopenia indicators so that patients with T2DM and sarcopenia can choose drugs more accurately. |
| 3 | **Objectives** | State specific objectives clearly, including pre-specified causal hypotheses (if any). State that MR is a method that, under specific assumptions, intends to estimate causal effects | 6-7 | In addition, the Mendelian randomized (MR) trial design provides a theoretical basis for elucidating the causal relationship between metformin and sarcopenia. |
|  | **METHODS** |  | 7 |  |
| 4 | **Study design and data sources** | Present key elements of the study design early in the article. Consider including a table listing sources of data for all phases of the study. For each data source contributing to the analysis, describe the following: | 7-8 | Figure1 and TableS2  Subsequently, drug target MR was employed to investigate the causal impact of metformin on various indicators related to sarcopenia. To accurately represent metformin's drug effect, cis-expression quantitative trait loci (eQTL) of downstream targets were utilized, providing insights into metformin's targeted genes that contribute to sarcopenia. |
|  | a) | Setting: Describe the study design and the underlying population, if possible. Describe the setting, locations, and relevant dates, including periods of recruitment, exposure, follow-up, and data collection, when available. | 8-9 | genome-wide association study (GWAS) data from UK Biobank (n = 1,366,167) and FinnGen (n = 218,007). |
|  | b) | Participants: Give the eligibility criteria, and the sources and methods of selection of participants. Report the sample size, and whether any power or sample size calculations were carried out prior to the main analysis | 8-9 | In genetic research, the Genome-Wide Association Study (GWAS) Catalog is a pivotal repository housing gene-phenotype associations data derived from an extensive published studies. The FinnGen Project and the UK Biobank are large-scale biomedical database to help us investigate the correlation between genomic information and health characteristics within a specific population. |
|  | c) | Describe measurement, quality control and selection of genetic variants | 9-11 | Firstly, exposure and outcome variables were investigated using GWAS data ,FinnGen Project and the UK Biobank . Secondly, instrumental variables strongly associated with exposure factors were selected, with a filtering condition set at a p-value <5e-08. Then, MR analysis was conducted and the results were visually displayed. Heterogeneity was assessed using the MR-Egger tests, with p-value < 0.05 indicating the presence of heterogeneity. |
|  | d) | For each exposure, outcome, and other relevant variables, describe methods of assessment and diagnostic criteria for diseases | 9 | Table2 |
|  | e) | Provide details of ethics committee approval and participant informed consent, if relevant | 8 | Ethical approval was not required for this study as all data sources relied upon publicly available datasets. Approval for the studies included in these datasets was obtained from the relevant institutional review boards. |
| 5 | **Assumptions** | Explicitly state the three core IV assumptions for the main analysis (relevance, independence and exclusion restriction) as well assumptions for any additional or sensitivity analysis | 10 | Heterogeneity analysis addresses the potential variability of instrumental variables stemming from diverse analysis platforms, experiments, populations, etc., thereby potentially influencing the outcomes of MR. The validation of the Inverse-Variance Weighted (IVW) algorithm involved various algorithms such as weighted median method. Leave-one sensitivity analysis aimed to assess the influence of each SNP on MR analysis outcomes. If outliers were found, they were systematically removed and the analysis was re-conducted. Additionally, pleiotropic analysis was conducted to ascertain whether the instrumental variable impacted the outcome through factors beyond the exposure variable.  Colocalization analysis is frequently utilized to determine whether two phenotypes are driven by the same causal variant in a specific genomic region, thereby strengthening the evidence of association[34-36]. Four hypotheses are typically considered in colocalization analysis, with the Bayesian method employed to calculate the posterior probability (PPH) values of these hypotheses. A standard criterion for screening shared SNP sites is that the PPH4 value ＞ 0.8. |
| 6 | **Statistical methods: main analysis** | Describe statistical methods and statistics used | 7-11 |  |
|  | a) | Describe how quantitative variables were handled in the analyses (i.e., scale, units, model) | 10 | exposure and outcome variables were from GWAS summary data , FinnGen Project and the UK Biobank |
|  | b) | Describe how genetic variants were handled in the analyses and, if applicable, how their weights were selected | 10 | instrumental variables strongly associated with exposure factors were selected, with a filtering condition set at a p-value <5e-08. |
|  | c) | Describe the MR estimator (e.g. two-stage least squares, Wald ratio) and related statistics. Detail the included covariates and, in case of two-sample MR, whether the same covariate set was used for adjustment in the two samples | 10 | Four hypotheses are typically considered in colocalization analysis, with the Bayesian method employed to calculate the posterior probability (PPH) values of these hypotheses. A standard criterion for screening shared SNP sites is that the PPH4 value ＞ 0.8. |
|  | d) | Explain how missing data were addressed | 11 | exposure and outcome variables were investigated using GWAS data ,FinnGen Project and the UK Biobank |
|  | e) | If applicable, indicate how multiple testing was addressed | 11 | Additionally, pleiotropic analysis was conducted to ascertain whether the instrumental variable impacted the outcome through factors beyond the exposure variable. This challenges the assumptions of independence and exclusivity. MR-Egger intercept test was utilized to detect pleiotropy and evaluate the robustness of results, with p < 0.05 indicating the presence of pleiotropy. |
| 7 | **Assessment of assumptions** | Describe any methods or prior knowledge used to assess the assumptions or justify their validity | 11 | Once a causal link between exposure and outcome is established, and a significant signal site is identified, it is imperative to elucidate the mechanism by which the site affects these two phenotypes. Colocalization analysis is frequently utilized to determine whether two phenotypes are driven by the same causal variant in a specific genomic region, thereby strengthening the evidence of association. |
| 8 | **Sensitivity analyses and additional analyses** | Describe any sensitivity analyses or additional analyses performed (e.g. comparison of effect estimates from different approaches, independent replication, bias analytic techniques, validation of instruments, simulations) | 11 | Table2  Heterogeneity and sensitivity analysis  Heterogeneity analysis addresses the potential variability of instrumental variables stemming from diverse analysis platforms, experiments, populations, etc., thereby potentially influencing the outcomes of MR. The validation of the Inverse-Variance Weighted (IVW) algorithm involved various algorithms such as weighted median method. Leave-one sensitivity analysis aimed to assess the influence of each SNP on MR analysis outcomes. If outliers were found, they were systematically removed and the analysis was re-conducted. Additionally, pleiotropic analysis was conducted to ascertain whether the instrumental variable impacted the outcome through factors beyond the exposure variable. This challenges the assumptions of independence and exclusivity. MR-Egger intercept test was utilized to detect pleiotropy and evaluate the robustness of results, with p < 0.05 indicating the presence of pleiotropy. |
| 9 | **Software and pre-registration** |  |  |  |
|  | a) | Name statistical software and package(s), including version and settings used | 11 | All of the analyses were implemented by R 4.3.3. |
|  | b) | State whether the study protocol and details were pre-registered (as well as when and where) | 7 | Research design is listed in page7. |
|  | **RESULTS** |  |  |  |
| 10 | **Descriptive data** |  |  |  |
|  | a) | Report the numbers of individuals at each stage of included studies and reasons for exclusion. Consider use of a flow diagram | 12-13 | Figure1 |
|  | b) | Report summary statistics for phenotypic exposure(s), outcome(s), and other relevant variables (e.g. means, SDs, proportions) | 15 | TableS2 |
|  | c) | If the data sources include meta-analyses of previous studies, provide the assessments of heterogeneity across these studies | 16 | Not applicable |
|  | d) | For two-sample MR:  i.  Provide justification of the similarity of the genetic variant-exposure associations between the exposure and outcome samples  ii.  Provide information on the number of individuals who overlap between the exposure and outcome studies | 15 | TableS2 |
| 11 | **Main results** |  |  |  |
|  | a) | Report the associations between genetic variant and exposure, and between genetic variant and outcome, preferably on an interpretable scale | 15 | Using samples from UK Biobank or FinnGen cohort, the effect of metformin targets on sarcopenia-related traits was investigated (Figure 3F)(Figure S1-S7). The result revealed that GDF15 expression was positively correlated with grip strength by using the IVW as gold standard, indicating a potential causal effect (OR = 1.010, 95% CI: 1.001-1.013, p = 0.024). Additionally, high expression of GDF15 was associated with high bone mineral density (OR = 1.050, 95% CI: 1.021-1.080, p < 0.001), as well as increased walk speed (OR = 1.021, 95% CI: 1.015-1.027, p < 0.001). Furthermore, GDF15 was found to be negatively correlated with osteoporosis (OR=0.455, 95%CI: 0.283-0.730, p=0.001), as well as death (HR = 0.990, 95% CI: 0.986-0.995, p < 0.001). While the correlation between GDF15 and lean body mass and fat-free mass did not reach statistical significance, there was a positive trend observed(Figure S2-S3), suggesting that GDF15, as a target of metformin, plays a significant role in reducing sarcopenia traits and associated risks |
|  | b) | Report MR estimates of the relationship between exposure and outcome, and the measures of uncertainty from the MR analysis, on an interpretable scale, such as odds ratio or relative risk per SD difference | 15 | Table S2 |
|  | c) | If relevant, consider translating estimates of relative risk into absolute risk for a meaningful time period | 15 | Not applicable |
|  | d) | Consider plots to visualize results (e.g. forest plot, scatterplot of associations between genetic variants and outcome versus between genetic variants and exposure) | 15 | Figure S1-12 |
| 12 | **Assessment of assumptions** |  |  |  |
|  | a) | Report the assessment of the validity of the assumptions | 15 | TableS2 |
|  | b) | Report any additional statistics (e.g., assessments of heterogeneity across genetic variants, such as *I^2^*, Q statistic or E-value) | 15 | Table2, Figure S1-12 |
| 13 | **Sensitivity analyses and additional analyses** |  |  |  |
|  | a) | Report any sensitivity analyses to assess the robustness of the main results to violations of the assumptions | 16 | Heterogeneity tests showed that there were no heterogeneity among these seven traits in Table S2. MR-Egger intercept term was utilized for the pleiotropy test, indicating the absence of horizontal pleiotropy (Table S2). |
|  | b) | Report results from other sensitivity analyses or additional analyses | 16 | A leave-one-out sensitivity analysis validated the robustness of the outcome.. |
|  | c) | Report any assessment of direction of causal relationship (e.g., bidirectional MR) | 16 | MR analysis indicated that increased GDF15 levels were associated with a positive causal effect on increased grip strength, bone mineral density, and walke pace. Additionally, elevated levels of GDF15 were linked to a detrimental impact on osteoporosis and death, indicating a beneficial feedback loop involving the metformin target GDF15 in muscle composition, skeletal muscle metabolism. |
|  | d) | When relevant, report and compare with estimates from non-MR analyses | 16 | **Metformin is significantly associated with risk of sarcopenia**  A scatter plot analysis was conducted to explore the correlation between days of medication and muscle mass, and revealed a positive correlation (r=0.217, p < 0.05) (Figure 3C). Furthermore, Kaplan-Meier (K-M)analysis demonstrated that patients using metformin had better survival rates than those not (HR=0.58, 95% CI: 0.51-0.63, p < 0.001) (Figure 3D). Subgroup analysis indicated similar results in non-sarcopenia patients (HR=0.61, 95% CI: 0.47-0.80, p < 0.001) (Figure 3E). Consistent with previous results, these findings suggest that metformin may be significant value in preventing sarcopenia in T2DM patients. |
|  | e) | Consider additional plots to visualize results (e.g., leave-one-out analyses) | 16 | Table2, Figure S1-12 |
|  | **DISCUSSION** |  |  |  |
| 14 | **Key results** | Summarize key results with reference to study objectives | 17-18 |  |
| 15 | **Limitations** | Discuss limitations of the study, taking into account the validity of the IV assumptions, other sources of potential bias, and imprecision. Discuss both direction and magnitude of any potential bias and any efforts to address them | 21 | Lastly, notable advantages were observed in the colocalization analysis, with a high posterior probability of shared causal variation (PPH4: exceeding the conventional threshold of 80%). However, several limitations should be noticed. Firstly, MR analysis may not fully capture the true magnitude of metformin's benefits. Secondly, the lack of information on metformin dosage in the NHANES database precluded an analysis of dose-response relationships.  Further Large-scale and multicenter studies are needed to substantiate our conclusions in the future. |
| 16 | **Interpretation** |  |  |  |
|  | a) | Meaning: Give a cautious overall interpretation of results in the context of their limitations and in comparison with other studies | 19 | The causal relationship between metformin use and sarcopenia has not yet been explored, we therefore conducted drug-targeted MR Analyses. The results revealed a significant negative association between high GDF15 expression and sarcopenia traits, as well as adverse events such as osteoporosis and death. |
|  | b) | Mechanism: Discuss underlying biological mechanisms that could drive a potential causal relationship between the investigated exposure and the outcome, and whether the gene-environment equivalence assumption is reasonable. Use causal language carefully, clarifying that IV estimates may provide causal effects only under certain assumptions | 19-20 | Furthermore, colocalization analysis identified a significant shared SNP site between eQTL expression of the GDF15 gene and the risk of sarcopenia, with a high Bayesian posterior probability verification of 99.9%, indicating a common genetic basis. Consistent with these findings, molecular docking analysis demonstrated robust interaction modes and binding energies between metformin and GDF15, suggesting a strong affinity between them. These findings collectively highlight the significant association between metformin use and a reduced risk of sarcopenia. Mechanistically, GDF15 may play an important role in metformin regulating the progression of sarcopenia. |
|  | c) | Clinical relevance: Discuss whether the results have clinical or public policy relevance, and to what extent they inform effect sizes of possible interventions | 20 | Our study found a significant association between metformin use and sarcopenia-related risk in T2D patients, which remained significant after adjusting for comorbidities such as smoking, alcohol consumption, education, and lifestyle factors. Previous research suggests that metformin may exert beneficial effects on age-related pathophysiology, particularly mechanisms related to energy utilization, which may impact skeletal muscle function. |
| 17 | **Generalizability** | Discuss the generalizability of the study results (a) to other populations, (b) across other exposure periods/timings, and (c) across other levels of exposure | 19-20 | This study had several innovative aspects. Firstly, it is the first comprehensive investigation to assess the impact of metformin on sarcopenia. Secondly, unlike previous studies, the independent genetic tools chosen for MR analysis were derived from large-scale GWAS with stringent quality control measures and association analysis thresholds. Moreover, the NHANES dataset was meticulously curated with strict inclusion and exclusion criteria, minimizing the influence of other medications on the primary study outcomes. |
|  | **OTHER INFORMATION** |  |  |  |
| 18 | **Funding** | Describe sources of funding and the role of funders in the present study and, if applicable, sources of funding for the databases and original study or studies on which the present study is based | 26 | Not applicable |
| 19 | **Data and data sharing** | Provide the data used to perform all analyses or report where and how the data can be accessed, and reference these sources in the article. Provide the statistical code needed to reproduce the results in the article, or report whether the code is publicly accessible and if so, where | 26 | The protocols of NHANES were approved by the institutional review board  of the National Center for Health Statistics, CDC (https://www.cdc.gov/nchs/  nhanes/irba98.htm). All participants provided written consent after being fully  informed. |
| 20 | **Conflicts of Interest** | All authors should declare all potential conflicts of interest | 26 | All authors should declare all potential conflicts of interest. |

This checklist is copyrighted by the Equator Network under the Creative Commons Attribution 3.0 Unported (CC BY 3.0) license.

1. Skrivankova VW, Richmond RC, Woolf BAR, Yarmolinsky J, Davies NM, Swanson SA, et al. Strengthening the Reporting of Observational Studies in Epidemiology using Mendelian Randomization (STROBE-MR) Statement. JAMA. 2021;under review.

2. Skrivankova VW, Richmond RC, Woolf BAR, Davies NM, Swanson SA, VanderWeele TJ, et al. Strengthening the Reporting of Observational Studies in Epidemiology using Mendelian Randomisation (STROBE-MR): Explanation and Elaboration. BMJ. 2021;375:n2233.
